# Predicting enzymatic reactions with a molecular transformer†

**DOI:** 10.1039/d1sc02362d

**Published:** 2021-05-25

**Authors:** David Kreutter, Philippe Schwaller, Jean-Louis Reymond

**Affiliations:** Department of Chemistry, Biochemistry and Pharmaceutical Sciences, University of Bern Freiestrasse 3 3012 Bern Switzerland jean-louis.reymond@dcb.unibe.ch; IBM Research Europe Säumerstrasse 4 8803 Rüschlikon Switzerland

## Abstract

The use of enzymes for organic synthesis allows for simplified, more economical and selective synthetic routes not accessible to conventional reagents. However, predicting whether a particular molecule might undergo a specific enzyme transformation is very difficult. Here we used multi-task transfer learning to train the molecular transformer, a sequence-to-sequence machine learning model, with one million reactions from the US Patent Office (USPTO) database combined with 32 181 enzymatic transformations annotated with a text description of the enzyme. The resulting enzymatic transformer model predicts the structure and stereochemistry of enzyme-catalyzed reaction products with remarkable accuracy. One of the key novelties is that we combined the reaction SMILES language of only 405 atomic tokens with thousands of human language tokens describing the enzymes, such that our enzymatic transformer not only learned to interpret SMILES, but also the natural language as used by human experts to describe enzymes and their mutations.

## Abstract 2

The enzymatic transformer was trained with a combination of patent reactions and biotransformations and predicts the structure and stereochemistry of enzyme-catalyzed reaction products with remarkable accuracy.

## Graphical abstract

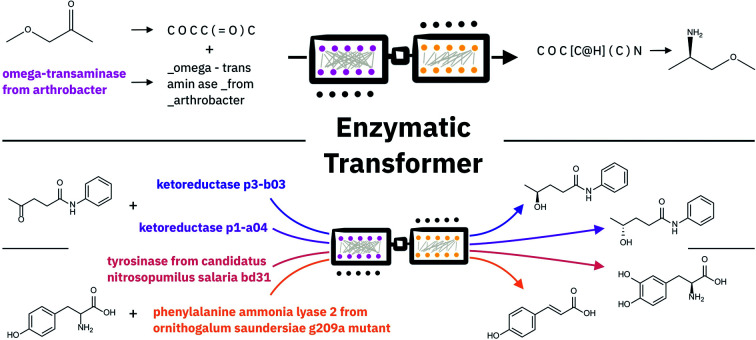

## Introduction

The use of enzymes for organic synthesis, commonly referred to as the field of biocatalysis, greatly contributes to organic synthesis methodology by providing the possibility to carry out highly chemo-, regio-, stereo- and enantio-selective transformations under mild and environmentally friendly conditions, often allowing the redesign and simplification of synthetic routes by enabling reactions that are not possible with conventional chemical reagents.^1,2^ The advent of directed enzyme evolution as a tool to increase enzyme performance has also greatly contributed to improve the range and efficiency of enzyme catalyzed reactions for organic synthesis.^3^ However, the implementation of biocatalytic steps in synthetic processes remains challenging because it is very difficult to predict whether a particular substrate might actually be converted by an enzyme to the desired product.

**Fig. 1:**
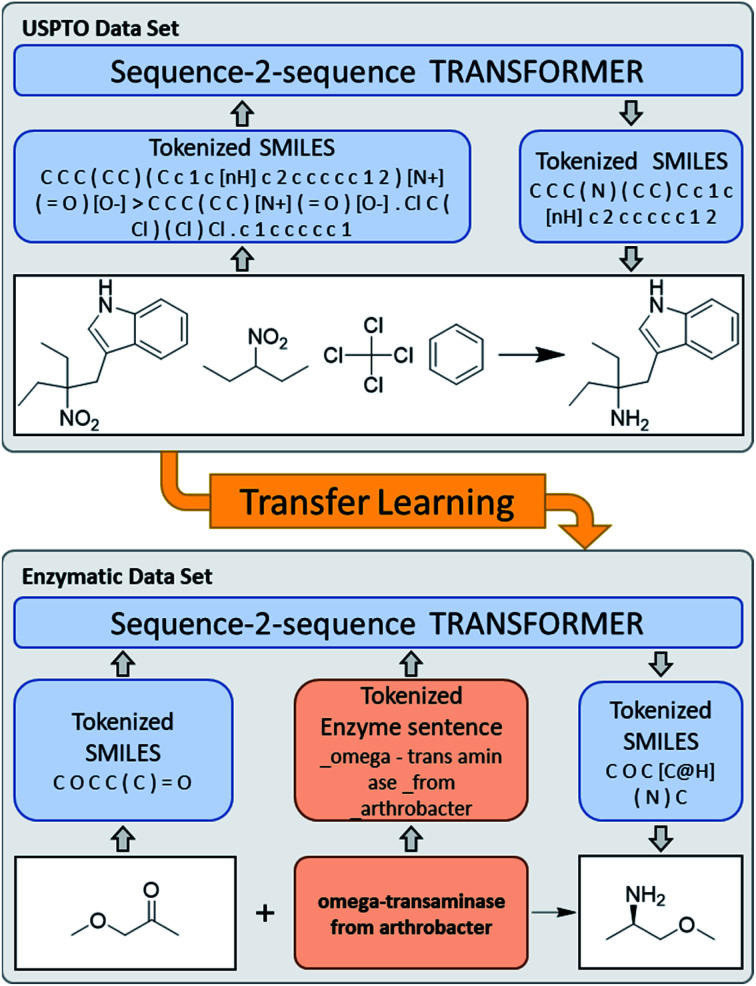
General concept of the enzymatic transformer training. The USPTO data set contains reactions SMILES describing reactants, reagents and products. The ENZR data set contains reaction SMILES as well as an additional text component.

Computer-assisted synthetic planning (CASP) comprises a range of artificial intelligence approaches to predict reaction products from reactant or reagents, or *vice versa*, and to plan retrosynthesis.^4–12^ Here we asked the question whether CASP might be exploited to predict the outcome of enzymatic reactions for organic synthesis. Recent efforts in predicting enzymatic reactions focused on metabolic reactions from the KEGG enzymatic reaction database and predictions of drug metabolism,^13–15^ as well as retrosynthetic planning with enzymatic reactions using a template based approach.^16^ Here we considered the molecular transformer,^17–19^ which is a sequence-to-sequence prediction model operating on text representations of reactions as reaction SMILES (Simplified Molecular Input Line Entry System)^20^ including stereochemistry. We set out to use multi-task transfer learning combining the USPTO dataset^21^ as a source of general chemistry knowledge with a few thousand enzymatic reactions collected from the scientific literature as a source of specialized knowledge (Fig. 1).

We used transfer learning previously to enable the molecular transformer to predict complex regio- and stereo-selective reactions at the example of carbohydrates.^22^ In this former study transfer learning was performed on a dataset of reactions described as SMILES, which are based on a vocabulary of only a few hundred atomic tokens identical to the vocabulary describing the general USPTO dataset used for primary training. One of the novelties of the present work on enzyme reactions is that we combine SMILES language for the substrates with human language for the enzyme descriptions. Those more diverse inputs result in an increase from 405 atomic tokens for SMILES only to a few thousand atomic and language tokens when describing enzyme reactions, implying that our transformer model had to learn to interpret not only the SMILES language but also natural language, as used by human experts to describe enzymes and their mutations.

## Result and discussion

### Reaction datasets

As a general chemistry dataset, we used the previously reported “USPTO stereo augmented” dataset derived from the patent mining work of Lowe, which contains, for each of the one million reactions in the USPTO dataset, the original reaction SMILES and a randomized SMILES version, both conserving stereochemical information.^23,24^ To compose a specialized dataset of enzymatic reactions, we extracted 70 096 reactions labeled as “enzymatic reactions” from the Reaxys database.^25^ We collected the data columns corresponding to reactant SMILES, product SMILES, and enzyme description (“reaction”, “reagent” and “catalyst”). Canonicalizing all SMILES and removing reactions lacking either reactants or products as well as duplicate entries (identical reactants, products and enzyme description) left 32 181 unique enzymatic reactions, each annotated with an enzyme description, referred to here as the ENZR dataset.

Although Reaxys does not cover the full spectrum of scientific literature about enzymes, the ENZR dataset contains a broad range of enzymes covering diverse reaction types, including not only highly specific enzymes such as glucose oxidases and dehydrogenases used in glucose monitoring devices,^26^ but also enzymes with a documented broad substrate scope for organic synthesis including mechanistically promiscuous enzymes,^27^ such as lipases used to promote aldol and Michael addition reactions,^28^ or ene-reductases capable of reducing oximes,^29^ thus providing a broad basis for training our model about the scope and specificity of different enzymes. We did not consider the enzyme databases KEGG^30^ or BRENDA^31^ because their data format is not homogeneous and many of the listed reactions are template-based and not assigned to documented examples.

**Fig. 2:**
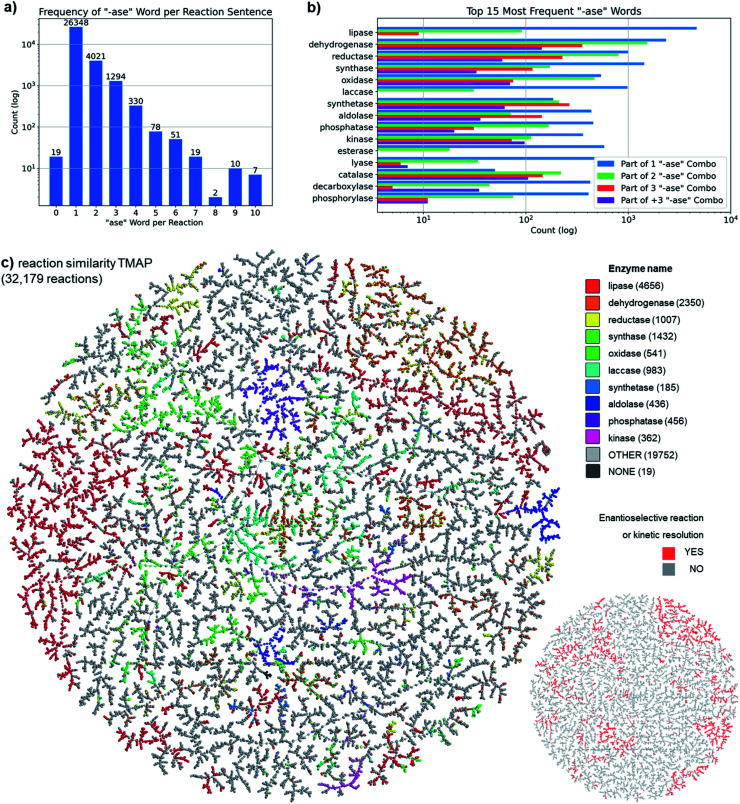
Analysis of the ENZR dataset. (a) Number of reactions depending on how many “-ase” words are present in the sentence. (b) Frequency of the top 15 “-ase” words depending on the count of enzyme name per reaction. (c) TMAP of reactions similarity color-coded by the 10 most frequent “-ase” words as listed in (b) combinations. The “other” category groups reactions with “-ase” words other than the top 10 “-ase” words as well as reactions containing more than one “-ase” word. Inset lower right: TMAP highlighting enantioselective and kinetic resolution reactions.

To better understand our ENZR dataset, we analyzed enzyme reactions in terms of the frequency of occurrence of words with the suffix “-ase”, which are the enzyme names, in the enzyme description. Across all enzyme reactions, 81.9% (26 348) contained a single “-ase” word, and 98.4% (31 663) contained one, two, or three “-ase” words (Fig. 2a). The largest group of single “-ase” word reactions involved a lipase (17%), a type of enzyme which is almost exclusively used alone. By contrast, dehydrogenases and reductases were most frequent in reactions involving two or more “-ase” words, reflecting that such enzymes are often used in processes involving enzyme-coupled cofactor regeneration systems. The ten most frequent “-ase” words corresponded to well-known enzyme families and together covered 50.3% of all enzyme reactions (the 15 most frequent “-ase” words covered 57.0% of all reactions, Fig. 2b). A finer analysis of enzyme families considering the complete enzyme description, which typically includes the enzyme source and the substrate type, showed that each enzyme family comprised a number of different enzymes (Fig. S1†).

To visualize our ENZR dataset, we used our recently reported TMAP (tree-map) algorithm, a powerful tool to represent very large high-dimensional datasets containing up to millions of datapoints as connected trees in two dimensions.^32^ In a first TMAP, we connected enzymatic reactions, each represented as a point, according to their similarity measured by the reaction fingerprint RXNFP, a recently reported reaction fingerprint derived from a neural network trained to classify patent chemical reactions.^33^ This analysis considered the transformation of substrates into product molecules but not the enzyme description in each ENZR entry. Color-coding the TMAP by the 10 most frequent “-ase” words mentioned above, corresponding to the most abundant enzyme families in the ENZR dataset, showed that these enzyme families formed relatively well separated clusters of reactions, illustrating that, similarly to organic reagents, enzymes carry out well-defined functional group transformations (Fig. 2c).

In a second color-coded version of the TMAP we labeled all enantioselective and kinetic resolution reactions, identified as reactions SMILES with no “@” characters in the reactants, indicating either the absence of chiral centers or an undefined stereochemistry at chiral centers, but the presence of at least one “@” character in the products SMILES, indicating a specific absolute configuration for chiral centers.^34^ This color-code showed that enantioselective and kinetic resolution reactions also formed defined clusters corresponding to biotransformations with mostly dehydrogenases, lipases and reductases (Fig. 2c, inset lower right).

The different enzymes also formed identifiable clusters in a different TMAP grouping reactions by substructure similarity of the reacting substrates using the extended connectivity fingerprint MHFP6 (Fig. S2†).^35^ This illustrated that enzymatic reactions in the ENZR dataset followed the well-known trend that enzymes only react with certain types of substrates, in contrast to chemical reagents which are usually only specific for functional groups. The range of substrates utilized by the enzymes covered a broad range of sizes from very small molecules such as pyruvate up to relatively large peptides (Fig. S2,† inset).

Taken together, the analysis above indicated that the ENZR dataset contained a diverse set of enzymatic reactions, with the expected biases towards the most frequently used enzymes in the field of biocatalysis such as lipases and dehydrogenases.

### Training and evaluation of transformer models for enzymatic reactions

Training a transformer model first requires tokenizing the input and output character strings to allow the model to learn which series of input tokens produces which series of output tokens. For the reaction SMILES in both USPTO and ENZR datasets, we used the approach reported previously for the general molecular transformer, which considers each character of the reaction SMILES as a separate token except Cl, Br, and character strings in square brackets, which denote special elements.^18^ The set of tokens necessary for describing reaction SMILES in the USPTO amounted to 405 so-called atomic tokens, and did not increase for describing the reaction SMILES portion of our ENZR dataset, which we first canonicalized using RDKit.^36^ To incorporate the enzyme information into our model, we tokenized the sentences describing the enzymes in the ENZR dataset using the Hugging Face Tokenizers library,^37^ which after preprocessing resulted in a vocabulary of 3004 atomic and language tokens to describe the ENZR dataset.

In view of evaluating transformer models, we split the USPTO stereo augmented dataset randomly into a training set (900 000 reactions, 90%, 1.8 million reactions after adding for each canonical training reaction a duplicate using non-canonical precursor SMILES), a validation and a test set (each 50 000 reactions, 5%).^24^ For the ENZR dataset, we first grouped reactions having the same product in different groups, and then split these groups into a training set (25 700 reactions, 80%), a validation and a test set (each 3200 reactions, 10%). Distributing these reaction groups rather than individual reactions into the different sets ensured that products which must be predicted in the validation or test sets have not been seen by the transformer during training or validation sets, respectively.

**Fig. 3:**
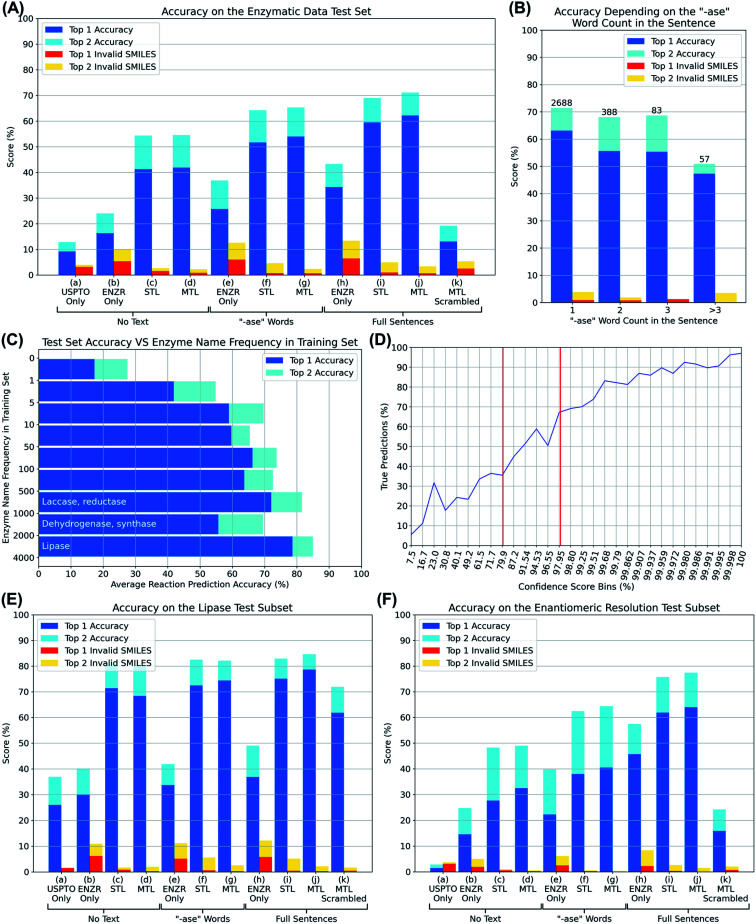
(A) Top prediction accuracy and invalid SMILES on the enzyme reaction test set for various models. (a) USPTO model from Schwaller *et al.* trained without any enzymatic transfer learning and tested without enzyme sentence. (b) Enzymatic DB without USPTO data set. (c) USPTO model transfer learned (sequential) to enzymatic DB trained without any enzyme description part. (d) USPTO model transfer learned (multi-task) to enzymatic DB trained without any enzyme description part. (e) Enzymatic DB without USPTO data set trained with ‘-ase’ words only. (f) USPTO model transfer learned (sequential) to enzymatic DB trained with ‘-ase’ words only. (g) USPTO model transfer learned (multi-task) to enzymatic DB trained with ‘-ase’ words only. (h) Enzymatic DB without USPTO data set trained with enzyme full sentences. (i) USPTO model transfer learned (sequential) to enzymatic DB trained with enzyme full sentences. (j) USPTO model transfer learned (multi-task) to enzymatic DB trained with enzyme full sentences. (k) Best multi-task model tested by swapping enzyme full sentences between reactions of the test set. (B) Accuracy on the test set depending on how many “-ase” words are present in the sentence. (C) Accuracy on the test set depending on how frequent the “-ase” words combination from the sentences appears in the training set. (D) True predictions rate against confidence scores, bins were adjusted to obtain an equal distribution of predictions over the bins. Vertical red bars represent our limits to indicate true or false predictions. (E) Top prediction accuracy and invalid SMILES on lipase reactions of the test set only. (F) Top prediction accuracy and invalid SMILES on enantiomeric resolution reactions of the test set only.

We then trained various models using OpenNMT^38^ and PyTorch,^39^ and evaluated them by presenting them with substrate SMILES, optionally together with the partial or full description of the enzyme, for each of the 3200 reactions in the test set. In each case, the model was challenged to write out the SMILES of the reaction product, including the correct stereochemistry, none of which had been seen by the model in the training or validation set. We analyzed whether the correct product was written out within the first one or first two solutions proposed by the model, as well as the percentage of invalid product SMILES, detected using RDKit, appearing among the first one or two solutions (top 1 and top 2 accuracy, blue and cyan bars, top 1 and top 2 invalid SMILES, red and orange bars, Fig. 3A).

We first evaluated if transformer models could be trained to predict reaction products from only the substrate by omitting any enzyme information during training. The UPSTO only model showed approximately 10% accuracy but a very low percentage of invalid SMILES, indicating that this model understood chemistry but lacked expertise in biotransformations (Fig. 3A, entry (a)). The ENZR only model also performed poorly (∼20% accuracy) and produced ∼10% invalid SMILES, reflecting that general chemistry training was insufficient with this relatively small dataset (Fig. 3A, entry (b)). Nevertheless, training with both models using sequential transfer learning (STL) or multi-task transfer learning (MTL) reached ∼50% accuracy, indicating that substrate structure was partially predictive of the outcome of enzymatic reactions even in the absence of any enzyme information (Fig. 3A, entries (c) and (d)). This partial prediction based on only the substrate reflects the fact that certain types of substrate molecules are only documented to react with specific enzymes in the ENZR dataset. For example, many alcohols are only documented to react with alcohol dehydrogenases to produce the corresponding ketone, such that a transformer model trained with the reaction SMILES learns to predict the ketone as the most likely product even without enzyme information, a prediction which is most of the time the correct one.

**Fig. 4:**
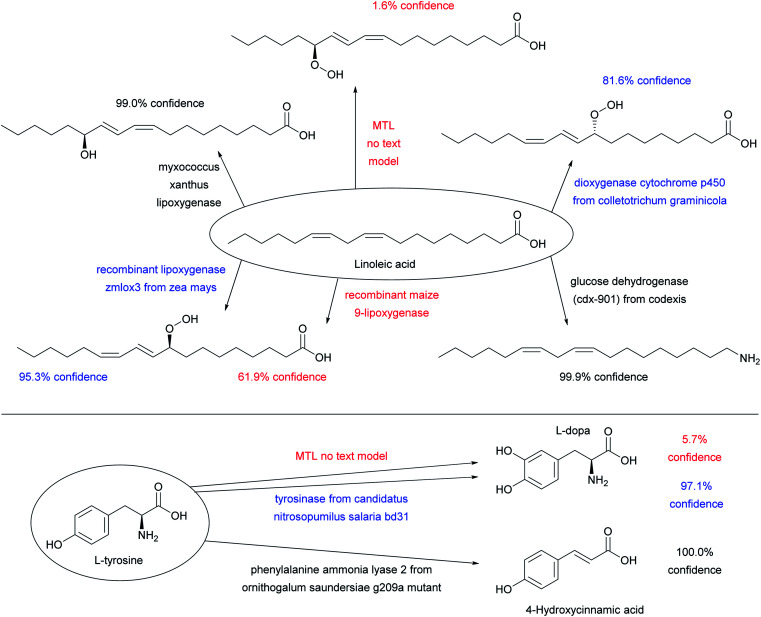
Examples of substrates applied to various enzymes using the MTL transformer with full sentences, which illustrate predictions of reactions from the test set not seen by the model during training. The color code indicates high confidence predictions (score > 98%, black), uncertain predictions (score 80–98%, blue), and low confidence predictions (score < 80%), see Fig. 3D for discussion of confidence scores. All enzymatic reactions are predicted correctly, however the confidence score varies. The predictions of the MTL no text model are shown to illustrate what the transformer predicts when the enzyme information is missing.

Adding enzyme information in form of “-ase” words alone did not significantly increase prediction performance when using only ENZR, however combining the data with the USPTO by transfer learning increased in terms of top 1 accuracy to 51.7% with STL and 54.0% with MTL (Fig. 3A, entries (e)–(g)). Top 1 prediction accuracy increased further up to 59.5% with STL and 62.2% with MTL when using the complete enzyme information as full sentence (Fig. 3A, entry (j)). Note that the model trained with ENZR alone only reached 34.3% top 1 accuracy with full enzyme names and produced ∼10% invalid SMILES, showing that the general chemistry training learned from USPTO was essential even with full enzyme information (Fig. 3A, entry (h)). Furthermore, testing the MTL with a test set in which the enzyme information was scrambled between reactions resulted in poor results (∼15% accuracy), indicating that the true enzyme information was required rather than the presence of random text information (Fig. 3A, entry (k)). Examples of the added value of enzyme information for predicting the outcome of an enzyme reaction are provided with the cases of linoleic acid conversion with various oxygenases and dehydrogenases, and the conversion of l-tyrosine by a lyase and a tyrosinase. These examples are taken from the test set and reflect true predictions since they have not been seen by the model during training or validation (Fig. 4).

### Analyzing the prediction performance of the enzymatic transformer

The comparisons above showed that an excellent prediction performance was reached by the transformer trained using MTL combining the USPTO and the ENZR dataset using full enzyme names as enzyme information. Retraining this model with different splits of training, validation and test sets gave indistinguishable results in terms of prediction accuracy. This model was selected for further investigation and is referred to as the “enzymatic transformer”.

Considering that many reactions in the ENZR dataset contain multiple enzymes, we wondered if our transformer might be confused in such situations because the main enzyme and the cofactor regeneration enzyme are not labeled as such. Indeed, the prediction accuracy of the enzymatic transformer was lower for reactions with multiple enzymes compared to reactions with a single enzyme (Fig. 3B). However, in many cases of multi-enzyme reactions including cofactor regeneration, the transformer provided the correct prediction when omitting the cofactor regenerating enzyme or swapping it for an equivalent one (glucose dehydrogenase to phosphite dehydrogenase, Fig. S3†).

Since transformer models require a large number of examples for good performance, we also tested prediction accuracy as function of the number of occurrences of the enzyme name in the training set. Indeed, a prediction accuracy of almost 80% was reached for lipases, which were the most abundant in the training set (Fig. 3C). Nevertheless, prediction accuracy reached a good level (∼60%) as soon as more than five examples of a particular enzyme were present in the training set.

In the best transformer model using MTL on full sentences, there was a clear association of the prediction confidence score with accuracy, as observed with other transformer models (Fig. 3D).^22^ Overall, 85.5% of the predictions with confidence score > 98% were true and 75.6% of the predictions with confidence score < 80% were false, suggesting to use confidence score values > 98% or <80% as indicators for a true (the reaction is worth testing) or false (the reaction outcome is uncertain) prediction.

Since the subset of the test set containing the word “lipase” performed best (Fig. 3C), we evaluated this subset exhaustively with all models (Fig. 3E). While models trained on the USPTO or ENZR dataset without enzyme information performed poorly (Fig. 3E, entries (a) and (b)), combining both sets with STL (entry (c)) or MTL (entry (d)) reached an excellent accuracy (>70%), indicating that the presence of an ester functional group is sufficient for the model to recognize a lipase biotransformation even in the absence of the enzyme name. However, models trained with ENZR alone using only the “ase” word or the full sentence performed poorly (Fig. 3E, entries (e) and (h)), showing that this relatively small dataset contained insufficient general chemistry knowledge to training even for the relatively simple lipase reaction. Overall, the model trained on both datasets using STL and the full enzyme description performed best for lipases, as observed in the entire dataset (Fig. 3E, entry (j)). However, scrambling the enzyme information between different reactions in the lipase only test set did not decrease prediction accuracy as dramatically as for the full set, reflecting the fact that all lipases catalyze very similar reactions. In addition, 36.89% of the lipase test set cases were reactions with *Candida antarctica* lipase B, the most frequently used lipase in biotranformations, in which case swapping the enzyme information does not induce any change.

Enzymatic reactions are often used to perform kinetic resolutions, typically using hydrolase enzymes such as lipases, or to transform achiral substrates into chiral products, typically to produce chiral alcohols or amines from achiral ketone precursors. To evaluate the performance of the transformer on such reactions, we defined enantiomeric resolutions as enzymatic reactions containing chiral centers, identified by the presence of at least one “@” character in the SMILES, in the reaction products only, which corresponded to 6495 reactions in the entire ENZR dataset (20.18%), and 687 reactions in the test set (21.35%). The relative performance of the different transformer models in this subset was comparable to that of the entire dataset, indicating that the transformer model was able to learn the enantiomeric preference of enantioselective enzymes as successfully as the overall enzymatic transformation (Fig. 3E).

### Examples of correct and incorrect predictions by the enzymatic transformer

**Fig. 5:**
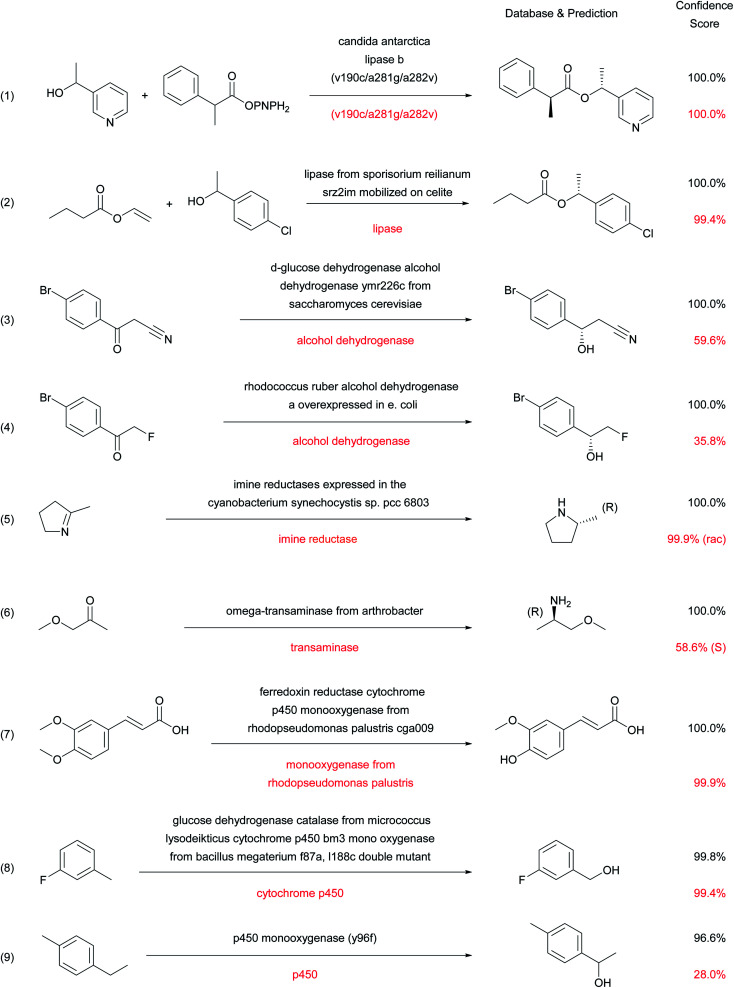
Examples of successful predictions by the enzymatic transformer.

The types of enzymatic reactions predicted correctly by the enzymatic transformer are well illustrated by selected cases (Fig. 5). These include the correct product prediction including chirality for kinetic resolutions using lipases (reactions (1)^40^ and (2)),^41^ two enantioselective reductions of ketones using alcohol dehydrogenases (reaction (3)^42^ and (4)),^43^ an enantioselective imine reduction (reaction (5))^44^ and reductive amination with a transaminase (reaction (6)).^45^

Considering that none of the products of these reactions have been seen by the model during training, the ability of the enzymatic transformer to predict not only the correct reaction product but also the correct stereochemical outcome of the enantiomeric resolution reactions is remarkable. It must be pointed out that the prediction is always done by analogy to examples, including cases of engineered enzymes. For instance, in reaction (1) with a mutant CALB enzyme, the transformer has learned from the training set that this triple mutant has an altered stereospecificity, and listing the mutation is sufficient for the model to make the correct prediction in the example from the test set. The product structure prediction is still correct but the stereoselectivity is lost when using simply “*Candida antarctica* lipase B” as enzyme description, which corresponds to the experimental result (Fig. S4†).

Cytochrome P450 mediated regioselective demethylation (reaction (7))^46^ or hydroxylations (reactions (8)^47^ and (9))^48^ further illustrate the predictive power of the enzymatic transformer. From the 405 cytochrome P450 mediated reactions in ENZR, 316 were used in the training set and 46 in the validation set. The resulting enzymatic transformer correctly predicted the product structure of 17 (40%) of the 43 cytochrome P450 reactions in the test set considering the top 1 predictions and 22 (51%) considering the top 2 predictions. The numbers increased to 21 (49%) correct predictions for the top 1 and 25 (58%) for the top 2 predictions when ignoring stereochemistry. These prediction accuracies are far from perfect but still very remarkable considering that the reaction site and type of cytochrome P450 reactions transformation are difficult to predict for a chemist (Fig. S5 and S6†).

In the above examples, a shorter description of the enzyme often reduces the confidence score and may induce errors in the predicted stereochemistry or product structure (red labels in Fig. 5 and S4†). Such errors when using short enzyme names are not surprising considering that models trained with only “-ase” words performed worse than models trained with the full enzyme description (Fig. 3A).

**Fig. 6:**
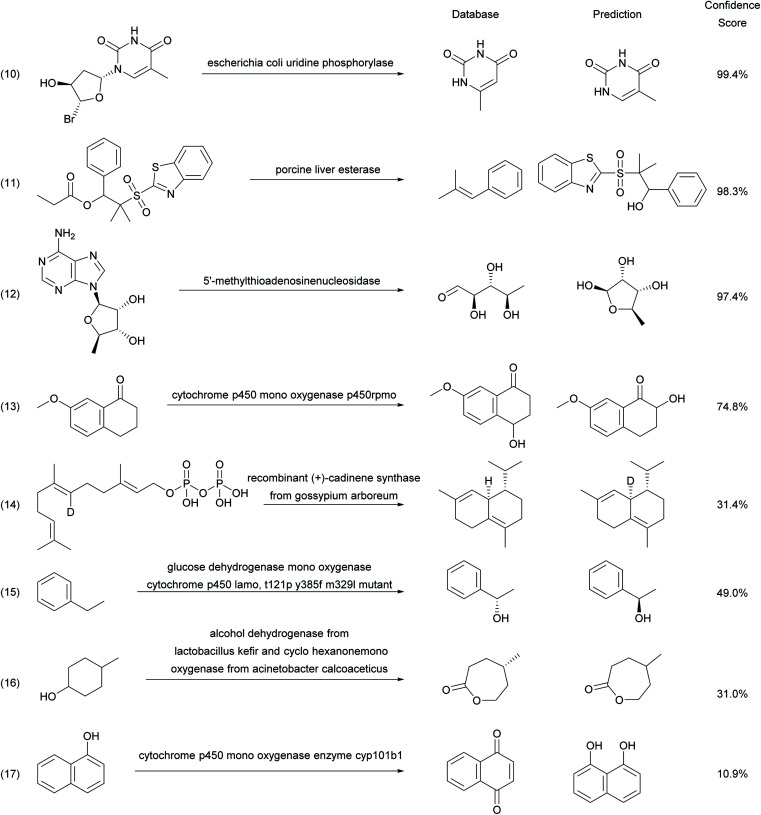
Examples of unsuccessful predictions by the enzymatic transformer.

Analyzing unsuccessful predictions by the enzymatic transformer in a random sample of 200 reactions from the test set selected to cover various reaction types and enzymes provides further insights (Fig. 6). Inaccurate predictions may sometimes simply reflect errors in database entries. For instance, the enzymatic transformer correctly predicts, with a high confidence score, the formation of thymine from the hydrolysis of a thymidine nucleoside analog by uridine phosphorylase, however the database entry wrongly recorded the isomeric 6-methyl-uracil as the product (reaction (10)).^49^ The model also correctly predicts with high confidence score the alcohol hydrolysis product in the hydrolysis of a β-hydroxysulfone by porcine liver esterase. However, this product is unstable and spontaneously eliminates to form a styrene, which is the product isolated and recorded in the database (reaction (11)).^50^ Furthermore, the model correctly predicts that 5-deoxy-*b*-d-ribofuranose is the product formed by the action of a nucleosidase on the parent adenosine nucleoside, which it writes down in the cyclic hemi-acetal form, while the database entry recorded the open-chain aldehyde form (reaction (12)).^51^

Other examples reflect true limitations of our model, for example errors in the regioselectivity of hydroxylation of 7-methoxy-3,4-dihydronaphthalen-1(2*H*)-one (reaction (13))^52^ and α-naphthol (reaction (17))^53^ by cytochrome P450. In the case of the formation of (+)-δ-cadinene from geranyl pyrophosphate by (+) cadinene synthase, our model predicts the correct product structure and stereochemistry, however the deuterium label, which is lost during cyclization, is wrongly incorporated into the predicted product (reaction (14)).^54^ The model may also predict the correct product structure but the opposite enantiomer, as illustrated for the benzylic hydroxylation of ethylbenzene by cytochrome P450 (reaction (15)),^55^ or with missing stereochemistry, as illustrated for the biotransformation of 4-methyl-cyclohexanol by a sequence of an alcohol dehydrogenase and a cyclohexanone monooxygenase to produce an enantiomerically pure lactone (reaction (16)).^56^

Note that the enzymatic transformer can only predict the structure of reaction products based on what it has learned from examples in the ENZR source database. For example, the reaction rates of 49 different alcohol substrates with a wild-type choline oxidase (WT) and an engineered version with an expanded substrate scope (M) have been reported with a broad range of values.^57^ However, the Reaxys entry used for ENZR attributed each reaction only to one of the two enzymes, which was in each case the faster reacting enzyme, even if the rates were almost equal. The enzymatic transformer was trained with a random subset of 32 reactions attributed to M and five reactions attributed to WT (Fig. S7†) and validated with five M and two WT cases (Fig. S8†). The model then correctly predicts the two WT and three M reactions in the test set, however in each case the same product is predicted with very high confidence for both WT and M enzymes (Fig. S9†). This prediction is correct for the two WT cases where the reported rates are almost equal for WT and M, but inaccurate for the three M cases where the activity of WT is much lower, including one case where even the M rate is impractically low, reflecting the fact that the training data does not consider reaction rate information.

### How to use the enzymatic transformer

The examples discussed above belong to the ENZR test set for which the product molecules have never been seen by the enzymatic transformer during training and validation, but they are recorded cases for which a look-up in the scientific literature will give the answer. In a possible application, one might use the enzymatic transformer to select which enzyme might be best suited for a given biotransformation not yet recorded in the dataset. To carry out such prediction, one would analyze the product structures and confidence scores returned by the model when presented with a given substrate and various enzymes.

**Fig. 7:**
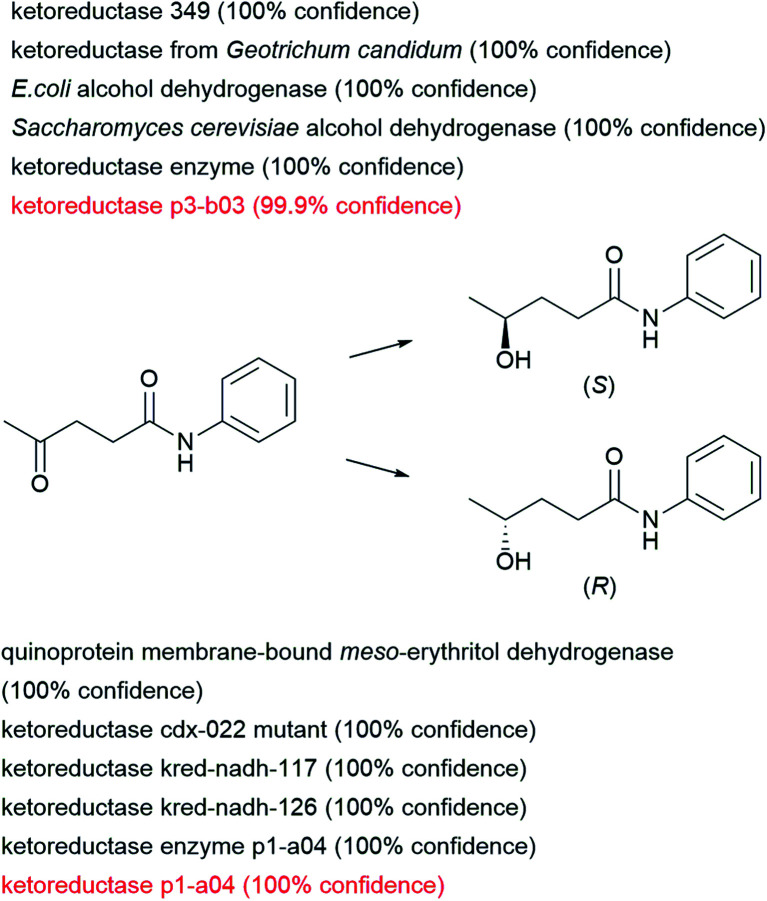
Examples of usage of the enzymatic prediction model to find suitable enzymes leading to different enantiomers. Screening sentences were extracted from the entire dataset. Filtering was applied for dehydrogenases and ketoreductases from single enzyme systems and filtered for simple sentences (less than 5 words). Resulting in a total of 223 sentences (163 dehydrogenases and 60 ketoreductases). Are shown the top 5 confidence score sentences leading to both enantiomers. Red colored sentences were present in the test set providing experimental proof.

As a theoretical example, we consider the reduction of levulinic anilide to either enantiomer of the corresponding chiral alcohol, a reaction which is not present in the training set. We used the enzymatic transformer to predict which product would be formed by exposing this ketone to 163 alcohol dehydrogenases and 60 ketoreductases in the ENZR dataset. In this case, the transformer model predicts with high confidence two experimentally verified cases of two different keto-reductases in the test set forming either the (*S*) or the (*R*) enantiomeric alcohol enantioselectively. In addition, the transformer also proposes high confidence reactions to either enantiomers involving other ketoreductase and alcohol dehydrogenases enzymes, which could be considered for experimental testing (Fig. 7).

One might also use the enzymatic transformer to predict which substrates might be converted by a given enzyme. To illustrate this point, we considered the enzyme “d-glucose dehydrogenase alcohol dehydrogenase ymr226c from *Saccharomyces cerevisiae*”, which is documented in six reactions of the training set to reduce various acetophenones enantioselectively and correctly predicts the product structure and stereochemistry for the 2 examples in the test set (Fig. S10,† substrates **D1** and **D2**). One can then challenge the enzymatic transformer to predict which product might be formed with further ketone substrates and the same enzyme. The transformer predicts the probably correct alcohol products with high confidence scores for ketones that are structurally related to the database examples (Fig. S10,† substrates **D3–D15**). Among further analogs that are less similar, three cases are predicted with high confidence (Fig. S10,† substrates **D16–D18**), and the remaining five cases have much lower confidence scores as well as sometimes unlikely product structure, indicating that the model is uncertain about the possible outcome of these reactions (Fig. S10,† substrates **D19–D22**).

## Conclusion

We had previously shown the principle of transfer learning to specialize the general USPTO transformer model at the example of carbohydrate reactions, however this approach used SMILES information only and a limited set of 405 tokens.^22^ Here we showed for the first time that the general USPTO transformer model can be used as a basis for transfer learning using a more complex language information, here an extended vocabulary of several thousand language and atomic tokens describing enzymatic reactions in text format. Despite of the relatively small size of the ENZR dataset of enzymatic reactions used here, the resulting enzymatic transformer model predicted the outcome of enzymatic transformations including enantioselective reactions with excellent accuracy. This type of approach might be extended in the future to incorporate additional information such as reaction conditions and experimental procedures.

It should be noted that the text descriptions of enzymes used in our ENZR dataset most often represent a rather plain description of the reaction and substrate involved, *e.g.* “tyrosine decarboxylase”, which provides a direct hint for the enzymatic transformer for proposing a product structure. Nevertheless, other descriptions of enzymes such as their EC number,^14^ their amino acid sequence or a representation of the sequence produced by an auto-encoder,^58,59^ might also be exploitable for the enzymatic transformer if these would be available since these descriptions in principle contain the same information, even if in a more indirect manner.^62^

Here we demonstrated the feasibility of using a text description of an enzyme to train a transformer model to predict product structure given a substrate and the enzyme. The same data type might be suitable to train a transformer to predict the substrate structure given a product and an enzyme (retro-synthesis) or to predict an enzyme name given a substrate and a product, however to succeed such models might require much larger datasets than the relatively small ENZR dataset used here.

In this study, we obtained the best prediction accuracies when using multi-task transfer learning based on the full description of the enzymes. However, model performance was limited by database size and was lower with enzymes for which only few examples were available. Furthermore, analysis of successes and failures showed that model performance is also limited by the occurrence of database entry errors. Model performance can probably be increased by using larger and higher quality training dataset. Furthermore, the performance of our enzymatic transformer model was highest with the enzymes that are most represented in the ENZR dataset, which were lipases and dehydrogenases due to the historical nature of the data source reflecting which enzymes have been mostly used in the literature. Considering that transformer models learn from example, increasing the performance for other types of biotransformations such as keto-reductases and monooxygenases will critically depend on acquiring training data for such types of enzymes. Provided the availability of experimental training data, the transfer learning approach demonstrated here should be optimally suited to integrate this data into predictive models capable of assisting chemists in implementing biotransformations for chemical synthesis.

## Methods

### Data collection

The USPTO data was downloaded from the patent mining work of Lowe.^24^ The ENZR data set was downloaded from Reaxys.^25^ Enzymatic reactions were found querying “enzymatic reaction” keywords directly in the search field.

### Transformer training

The enzymatic transformer model was trained based on the molecular transformer work from Schwaller *et al.*^18^ The version 1.1.1 of OpenNMT,^38^ freely available on GitHub,^60^ were used to preprocess, train and test the models. Minor changes were performed based on the version of Schwaller *et al.*^18^ SMILES were also tokenized using the same tokenizer as Schwaller *et al.*^18^ The ENZR description sentences were tokenized by the Hugging Face Tokenizers^37^ using a byte pair encoding^61^ resulting in a vocabulary of 6139 language tokens (top 40 most frequent tokens in Fig. S11†) for which the occurrence frequencies follow a power-law distribution shown in Fig. S12.† For our model, we used the 3000 most frequent tokens representing 97.4% of tokens found in ENZR sentences. The 3139 remaining tokens only represent 2.6% of occurrences and have less important frequencies going from 7 to 1. The following hyperparameters were used for the multi-task model:

preprocess.py -train_ids ENZR ST_sep_aug

-train_src $DB/ENZR/src_train.txt $DB/ST_sep_aug/src-train.txt

-train_tgt $DB/ENZR/tgt_train.txt $DB/ST_sep_aug/tgt-train.txt

-valid_src $DB/ENZR/src_val.txt -valid_tgt $DB/ENZR/tgt_val.txt

-save_data $DB/Preprocessed

-src_seq_length 3000 -tgt_seq_length 3000

-src_vocab_size 3000 -tgt_vocab_size 3000

-share_vocab -lower

train.py -data $DB/Preprocessed

-save_model ENZR_MTL -seed 42 -train_steps 200000 -param_init 0

-param_init_glorot -max_generator_batches 32 -batch_size 6144

-batch_type tokens -normalization tokens -max_grad_norm 0 -accum_count 4

-optim adam -adam_beta1 0.9 -adam_beta2 0.998 -decay_method noam

-warmup_steps 8000 -learning_rate 4 -label_smoothing 0.0 -layers 4

-rnn_size 384 -word_vec_size 384

-encoder_type transformer -decoder_type transformer

-dropout 0.1 -position_encoding -global_attention general

-global_attention_function softmax -self_attn_type scaled-dot

-heads 8 -transformer_ff 2048

-data_ids ENZR ST_sep_aug -data_weights 1 9

-valid_steps 5000 -valid_batch_size 4 -early_stopping_criteria accuracy

### Validation

Canonicalized SMILES were compared to assess the accuracy of the models. Distribution of the training, validation and test set was randomly distributed after being grouped by reaction product multiple time resulting in constant accuracy.

### TMAPs

TMAPs were computed using standard parameters.^32^ The reaction fingerprint (RXNFP)^33^ as well as the molecular substructure fingerprint (MHFP6)^35^ was computed with a dimension of 256.

## Availability of data and materials

The USPTO data is available from the patent mining work of Lowe.^24^ Reactions from Reaxys are accessible with subscription. The modified version of OpenNMT as well as the code for data extraction and preprocessing as well as to tokenize, train and test the model are available from: https://github.com/reymond-group/OpenNMT-py.

## Author contributions

DK designed and carried out the study and wrote the paper, PS provided support on the transformer model and wrote the paper, JLR designed and supervised the study and wrote the paper.

## Conflicts of interest

The authors declare that they have no competing interests.

## Supplementary Material

SC-012-D1SC02362D-s001
