# Supplementary material for: Predicting enzymatic reactions with a molecular transformer
Source: Chem Sci. 2021 May 25;12(25):8648–59. doi: 10.1039/d1sc02362d (PMC8246114; doi:10.1039/d1sc02362d)
Supplement: SC-012-D1SC02362D-s001 [file SC-012-D1SC02362D-s001.pdf]

Electronic Supplementary Material (ESI) for Chemical Science.
This journal is © The Royal Society of Chemistry 2021

Supporting Information:

Predicting Enzymatic Reactions with a Molecular Transformer

David Kreutter,[a] Philippe Schwaller[a), b)] and Jean-Louis Reymond[a)]*

[a)] Department of Chemistry and Biochemistry, University of Bern, Freiestrasse 3, 3012 Bern, Switzerland; [b)] IBM Research, Europe, Säumerstrasse 4, 8803 Rüschlikon, Switzerland

e-mail: jean-louis.reymond@dcb.unibe.ch

Dehydrogenase frequency analysis

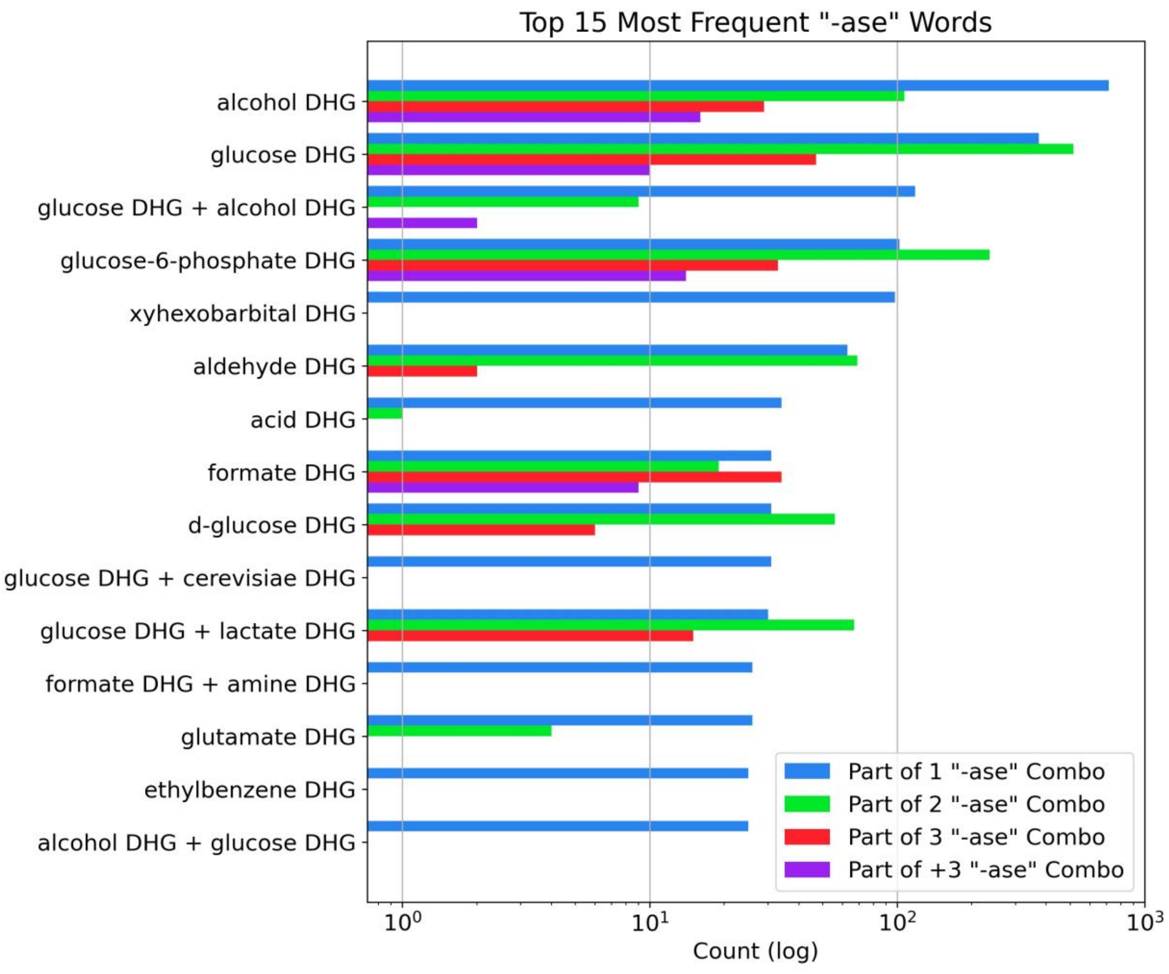

Figure S1. Analysis of the dehydrogenase ("DHG") diversity in the entire ENZR dataset.

TMAP of the ENZR dataset by substrate similarity

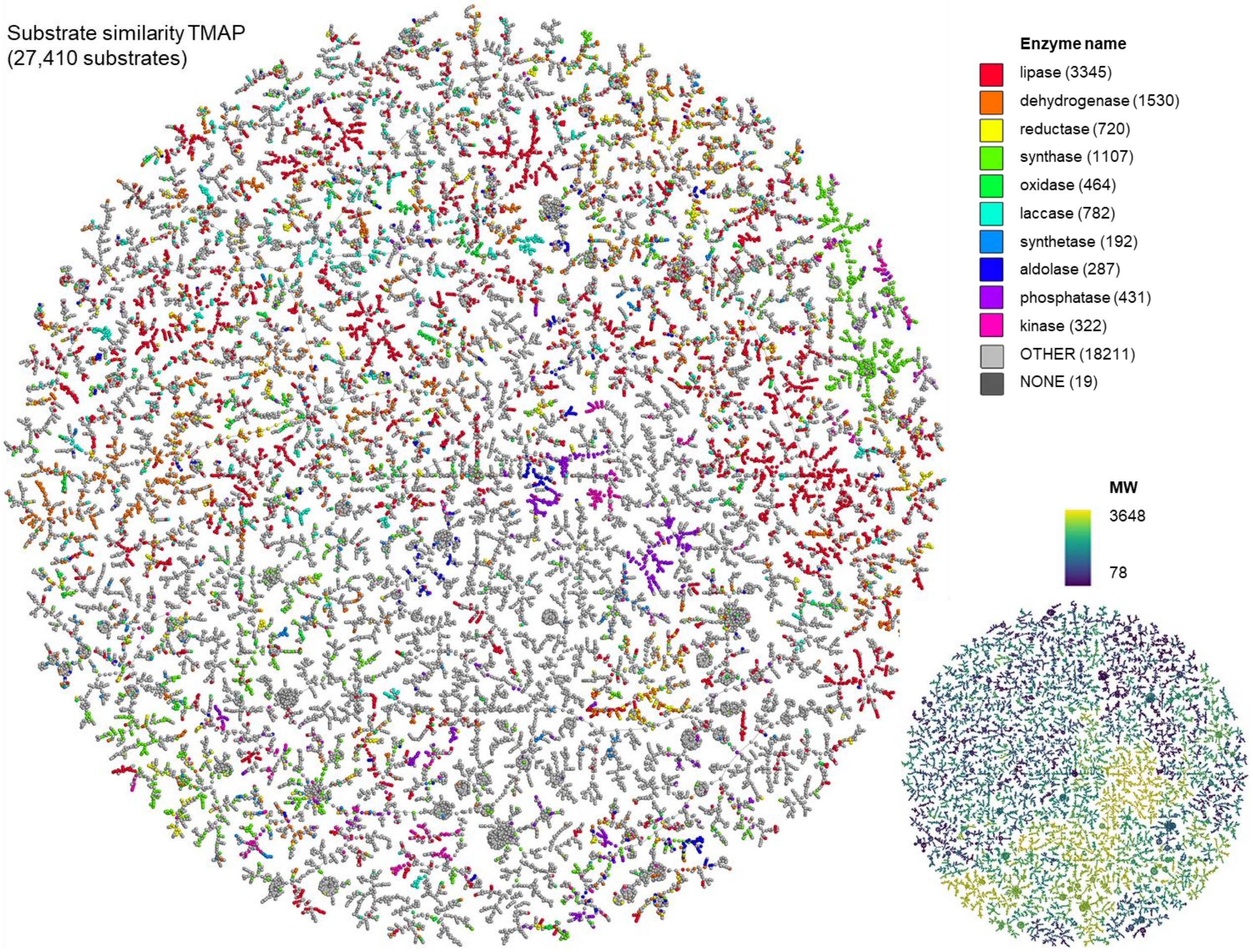

Figure S2. TMAP of the ENZR dataset analyzed by substrate similarity and color-coded by "-ase" word combinations. Inset: TMAP color-coded by substrate molecular weight.

Cofactor importance in the prediction.

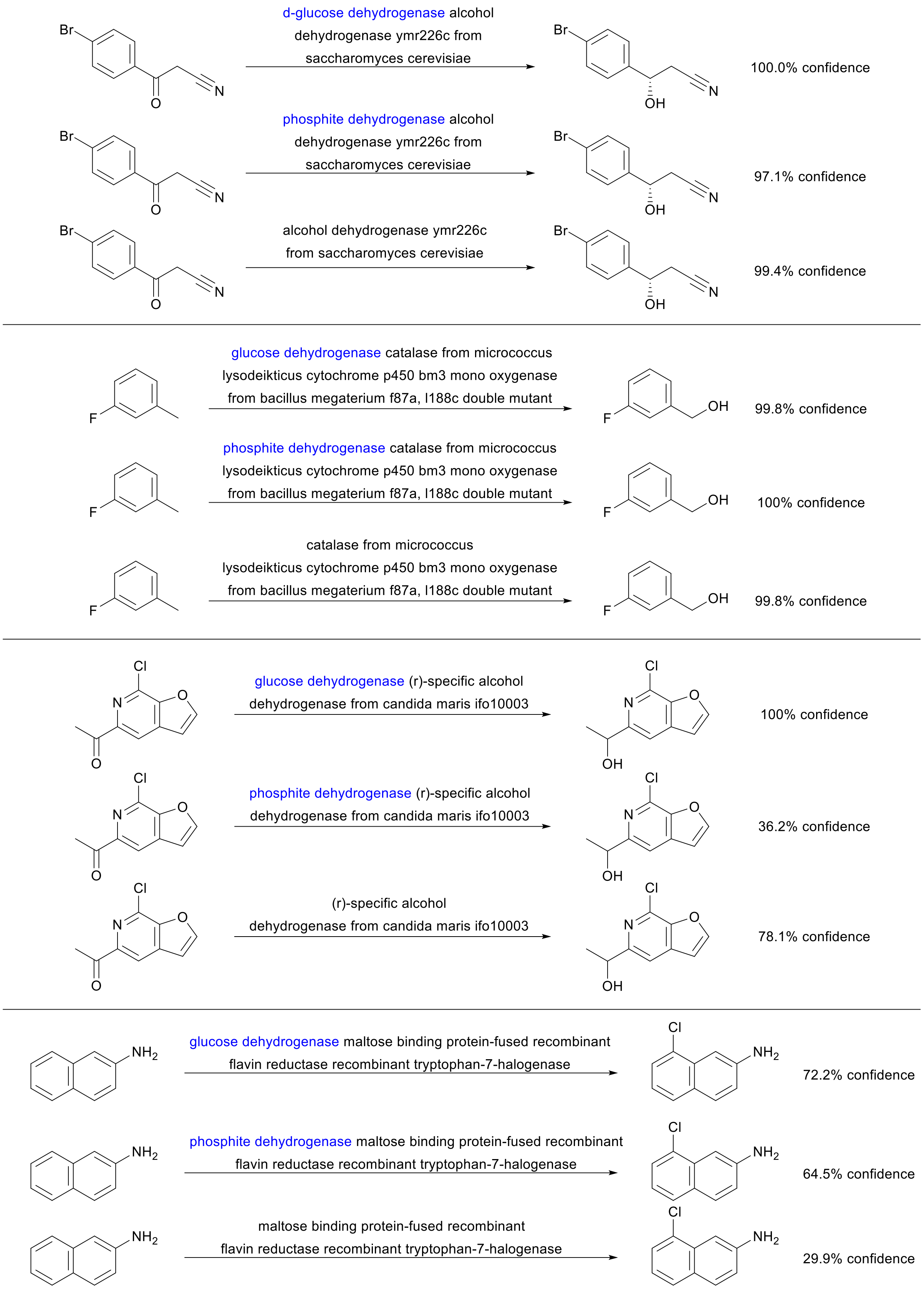

Figure S3. Examples of cofactor generator swapped or removed.

Effect of word on the prediction.

Entry	Reaction (Database / Prediction 1)	Conditions	Confidence Score	Rank
(1)	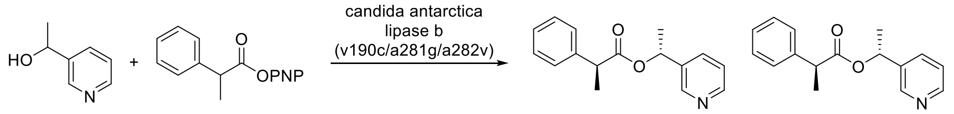	candida antarctica lipase b (v190c/a281g/a282v)	100.0%	1
(1a)	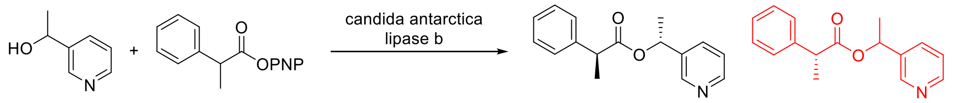	candida antarctica lipase b	13.4%	3
(1b)	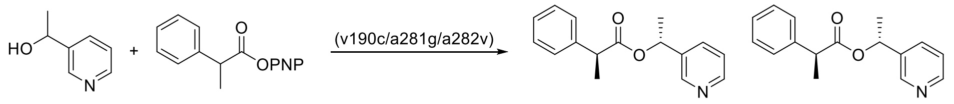	(v190c/a281g/a282v)	100.0%	1
(1c)	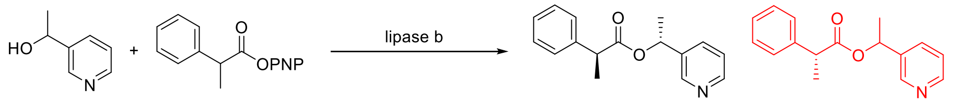	lipase b	5.6%	0

Entry	Reaction (Database / Prediction 1)	Conditions	Confidence Score	Rank
(2)	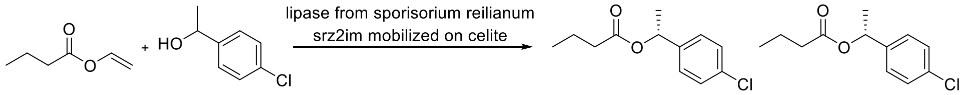	lipase from sporisorium reilianum srz2im mobilized on celite	100.0%	1
(2a)	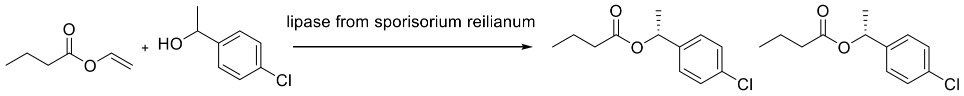	lipase from sporisorium reilianum	100.0%	1
(2b)	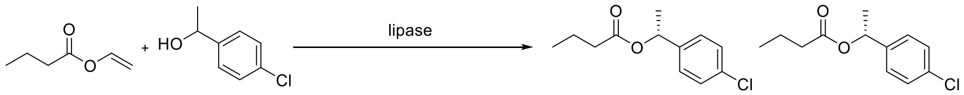	lipase	99.4%	1

Entry	Reaction (Database / Prediction 1)	Conditions	Confidence Score	Rank
(3)	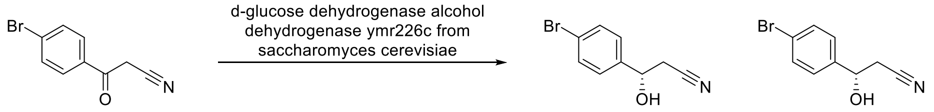	d-glucose dehydrogenase alcohol dehydrogenase ymr226c from saccharomyces cerevisiae	100.0%	1
(3a)	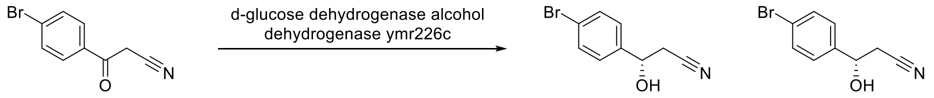	d-glucose dehydrogenase alcohol dehydrogenase ymr226c	100.0%	1
(3b)	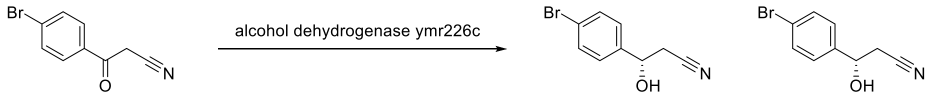	alcohol dehydrogenase ymr226c	100.0%	1
(3c)	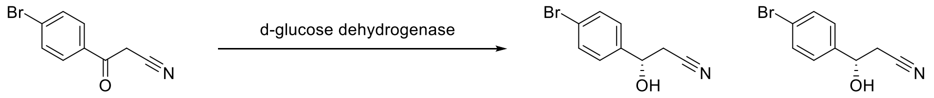	d-glucose dehydrogenase	92.6%	1
(3d)	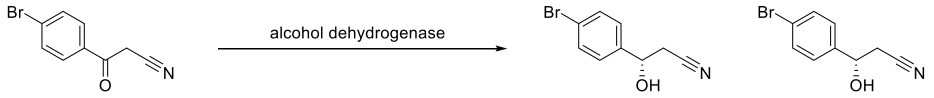	alcohol dehydrogenase	59.6%	1
(3e)	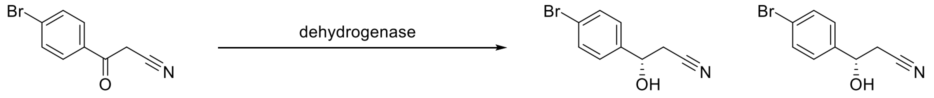	dehydrogenase	41.5%	1

	Substrate	Reagent	Database	Prediction 1	Confidence Score	Rank
(4)	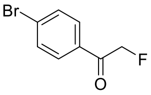	rhodococcus ruber alcohol dehydrogenase a overexpressed in e. coli	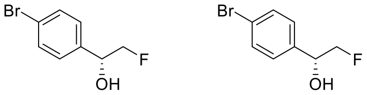	(see left)	100.0%	1
(4a)	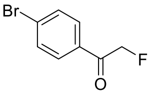	alcohol dehydrogenase a overexpressed in e. coli	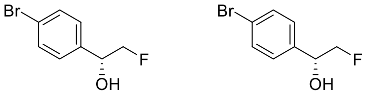	(see left)	100.0%	1
(4b)	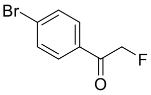	rhodococcus ruber alcohol dehydrogenase	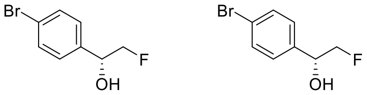	(see left)	26.1%	1
(4c)	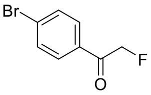	alcohol dehydrogenase	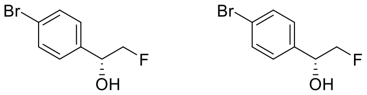	(see left)	35.8%	1
(4d)	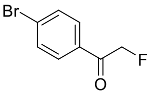	dehydrogenase	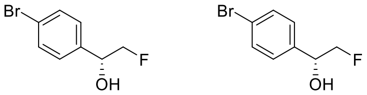	(see left)	32.0%	1

	Substrate	Reagent	Database	Prediction 1	Confidence Score (Pred1)	Prediction 2	Confidence Score (Pred2)	Rank
(5)	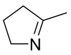	imine reductase s expressed in the cyanobacterium synechocystis sp. pcc 6803	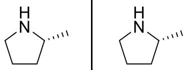	(see left)	100.0%	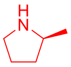	0.0%	1
(5a)	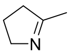	imine reductase s expressed in the cyanobacterium synechocystis	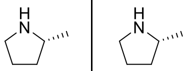	(see left)	100.0%	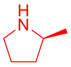	0.0%	1
(5b)	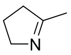	imine reductase s expressed	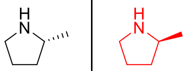	(see left)	22.3%	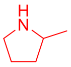	14.3%	3
(5c)	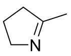	imine reductase s	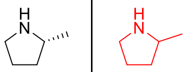	(see left)	99.9%	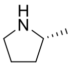	0.0%	2
(5d)	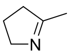	imine reductase	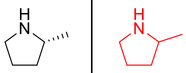	(see left)	99.9%	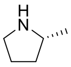	0.0%	2
(5e)	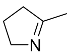	reductase	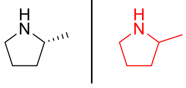	(see left)	99.8%	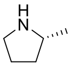	0.0%	2

	Substrate	Reagent	Database	Prediction 1	Confidence Score (Pred1)	Prediction 2	Confidence Score (Pred2)	Rank
(6)	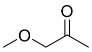	omega-transaminase from arthrobacter	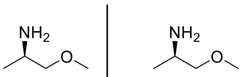	(see left)	100.0%	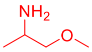	0.0%	1
(6a)	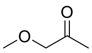	omega-transaminase	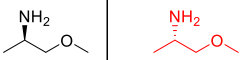	(see left)	99.0%	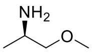	0.0%	2
(6b)	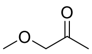	transaminase	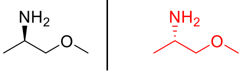	(see left)	58.6%	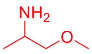	2.6%	3

	Reaction	Enzyme	Database / Prediction	Confidence Score	Rank
(7)	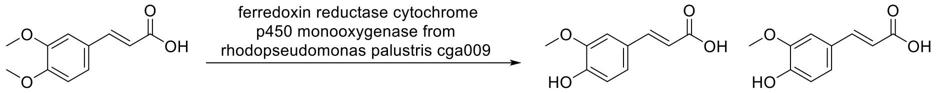	ferredoxin reductase cytochrome p450 monooxygenase from rhodopseudomonas palustris cga009		100.0%	1
(7a)	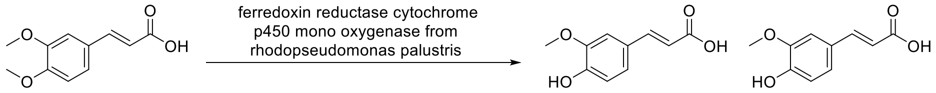	ferredoxin reductase cytochrome p450 mono oxygenase from rhodopseudomonas palustris		99.8%	1
(7b)	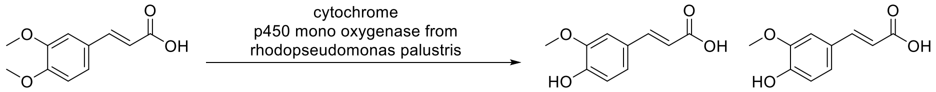	cytochrome p450 mono oxygenase from rhodopseudomonas palustris		99.8%	1
(7c)	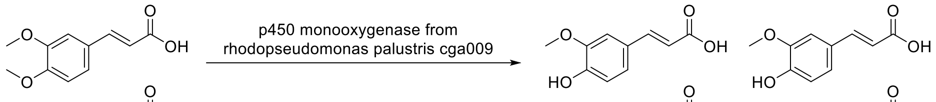	p450 monooxygenase from rhodopseudomonas palustris cga009		99.8%	1
(7d)	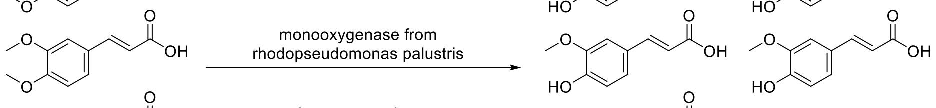	monooxygenase from rhodopseudomonas palustris		99.9%	1
(7e)	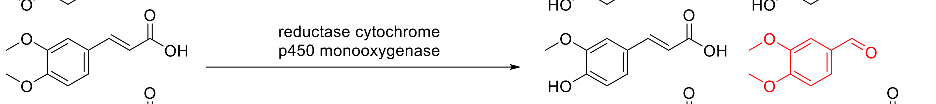	reductase cytochrome p450 monooxygenase		43.0%	0
(7f)	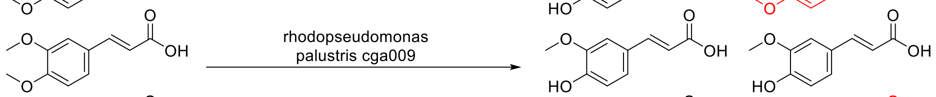	rhodopseudomonas palustris cga009		100.0%	1
(7g)	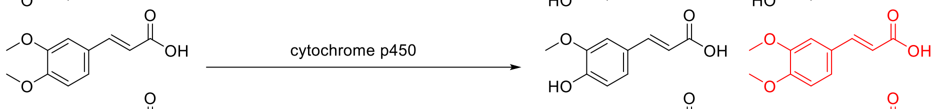	cytochrome p450		16.4%	0
(7h)	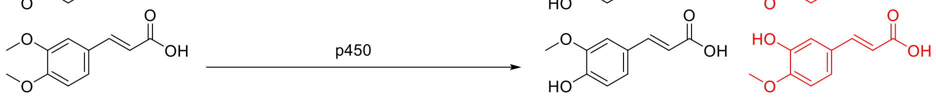	p450		31.5%	0

	Reaction	Enzyme	Database / Prediction	Confidence Score	Rank
(8)	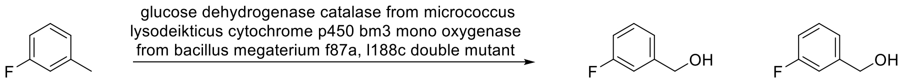	glucose dehydrogenase catalase from micrococcus lysodeikticus cytochrome p450 bm3 mono oxygenase from bacillus megaterium f87a, I188c double mutant		99.8%	1
(8a)	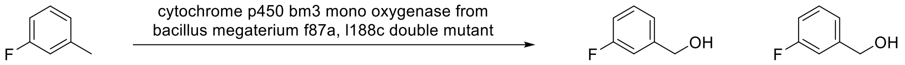	cytochrome p450 bm3 mono oxygenase from bacillus megaterium f87a, I188c double mutant		99.6%	1
(8b)	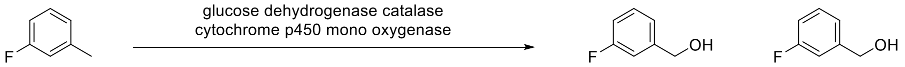	glucose dehydrogenase catalase cytochrome p450 mono oxygenase		100.0%	1
(8c)	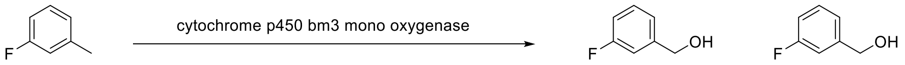	cytochrome p450 bm3 mono oxygenase		37.8%	1
(8d)	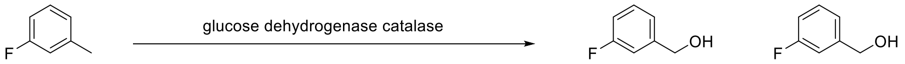	glucose dehydrogenase catalase		97.0%	1
(8e)	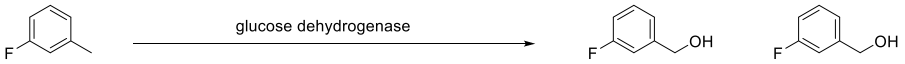	glucose dehydrogenase		61.2%	1
(8f)	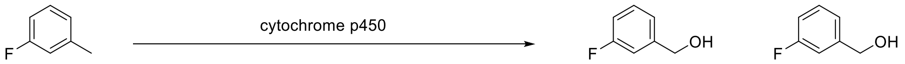	cytochrome p450		99.4%	1

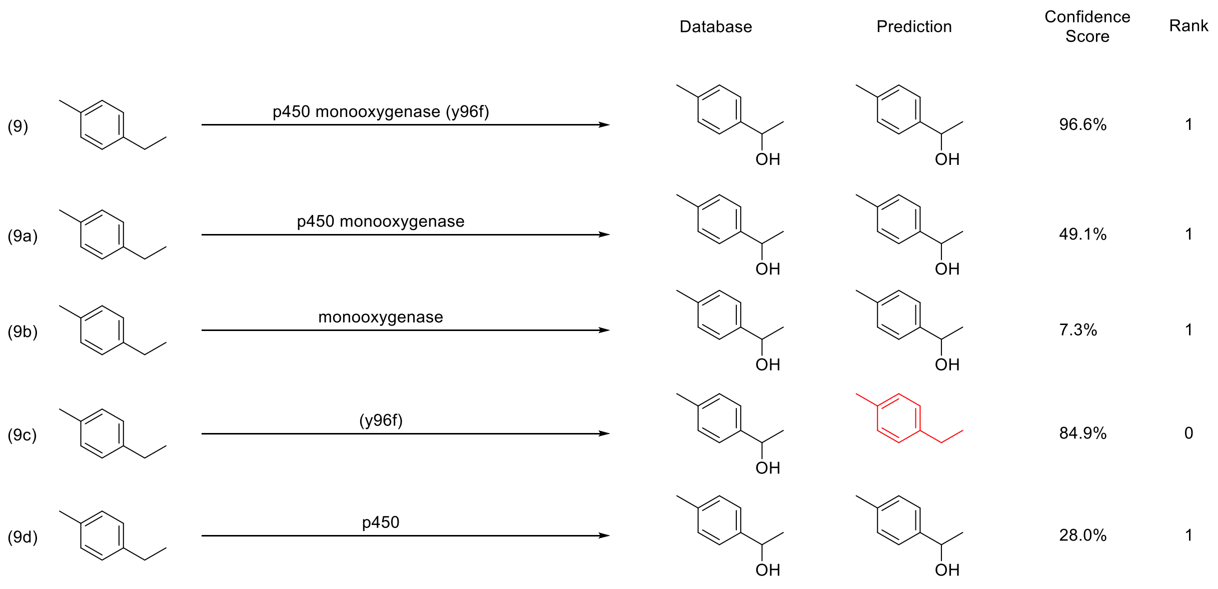

Figure S4. Examples of predictions from success examples from figure 4 with a variety of truncated sentences. “Rank” represent the top position prediction containing the correct product.

All P450 reactions from the test set.

Substrate	Enzymes	Database & Prediction	Confidence Score
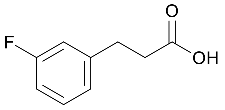	epoxidehydrolase from aspergillus niger p450 peroxygenase oletje formate dehydrogenase mutant from candida bodinii p450 monoxygenase l75v-v78f-f87v-l181f-l262v-a328f-l437v-t438f mutant	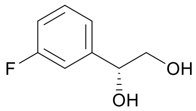	100.0%
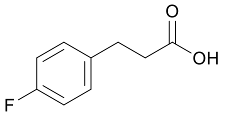	phosphite dehydrogenase oletje decarboxylase p450bm3 reductase domain fusion protein catalase	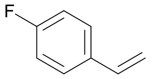	100.0%
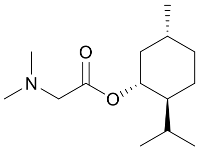	glucose-6-phosphate dehydrogenase cytochrome p450 monooxygenase pikc d50n d176q e246a mutant enzyme fused to rhfred reductase	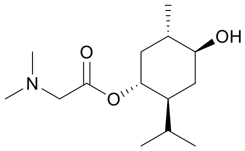	100.0%
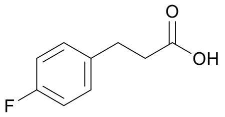	p450 peroxygenase oletje	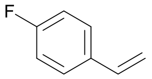	100.0%
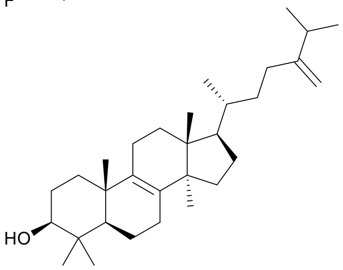	isocitrate dehydrogenase aspergillus fumigatus afcpr1-q4wm67 cytochrome p450 reductase recombinant acanthamoeba castellanii sterol c14-demethylase cyp51	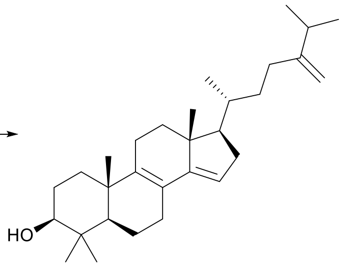	100.0%
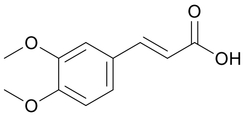	ferredoxin reductase cytochrome p450 monooxygenase from rhodopseudomonas palustris cga009	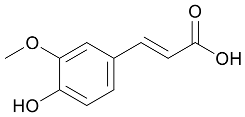	100.0%
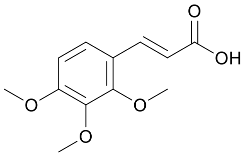	ferredoxin reductase cytochrome p450 monooxygenase from rhodopseudomonas palustris cga009	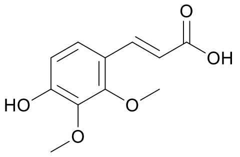	99.9%
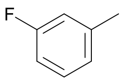	glucose dehydrogenase catalase from micrococcus lysodeikticus cytochrome p450 bm3monooxygenase from bacillus megaterium f87a, l188c double mutant	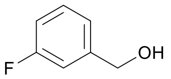	99.8%
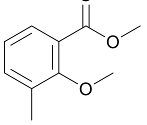	glucose dehydrogenase catalase from micrococcus lysodeikticus cytochrome p450 bm3monooxygenase from bacillus megaterium f87a, l188c double mutant	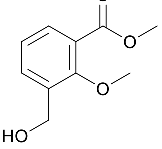	99.5%
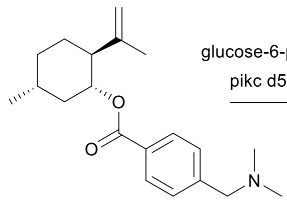	glucose-6-phosphate dehydrogenase cytochrome p450 monooxygenase pikc d50n d176q e246a mutant enzyme fused to rhfred reductase	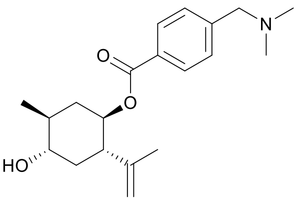	99.4%

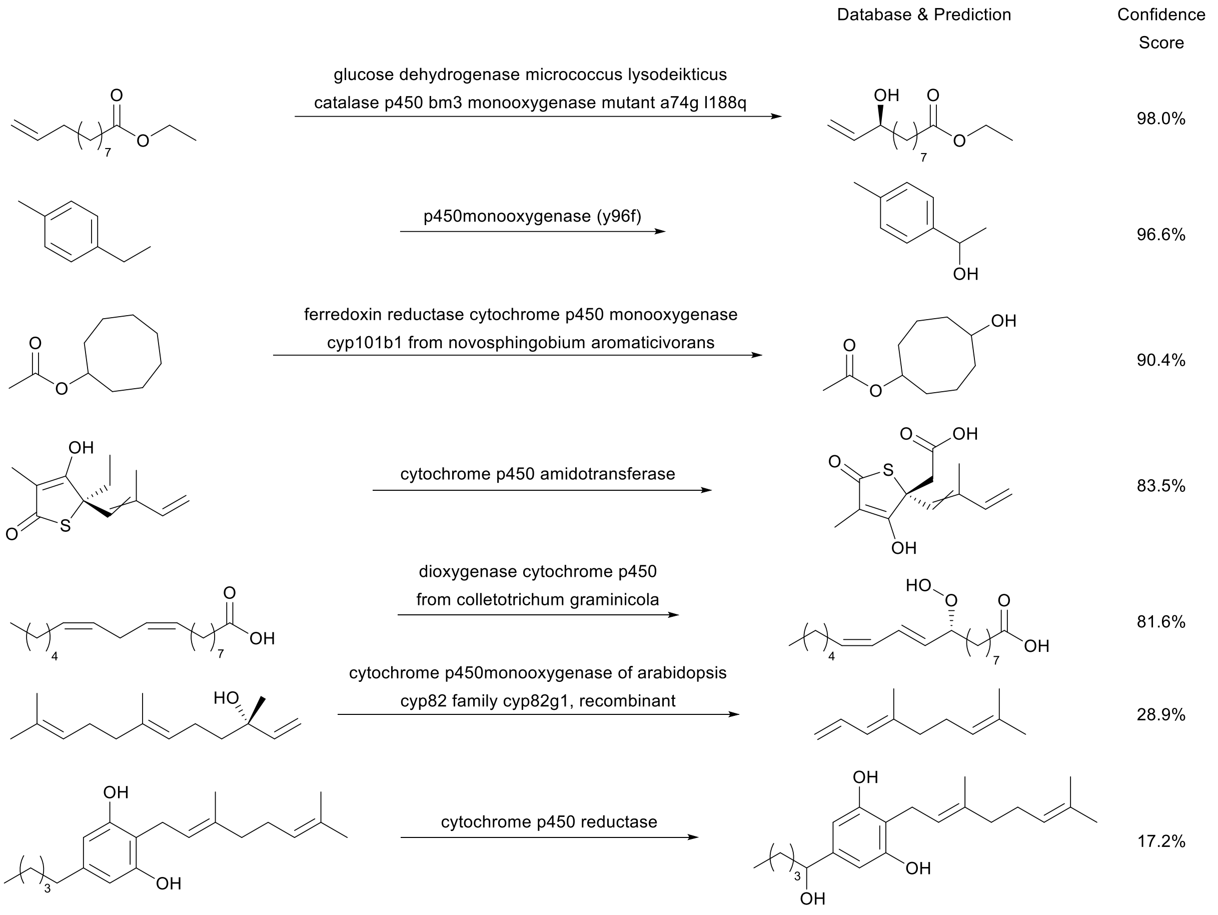

Figure S5. Every reaction from the test set containing “p450” in the sentence correctly predicted by the full sentence model. Reactions sorted by decreasing confidence score.

		Database	Top 1 Prediction	Confidence Score	Rank
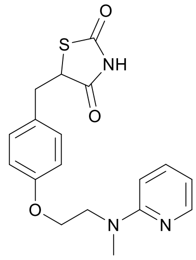	cytochrome p450 reductase	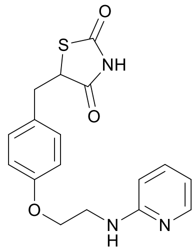	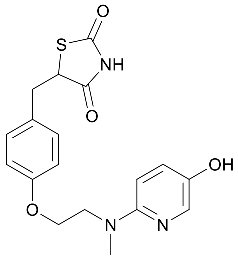	98.1%	0
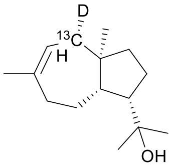	cytochrome p450monooxygenase cyp267b1 from sorangium cellulosum	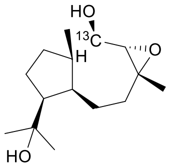	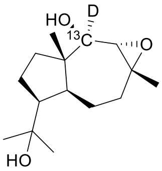	96.1%	0
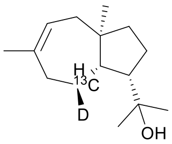	cytochrome p450monooxygenase cyp267b1 from sorangium cellulosum	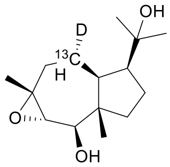	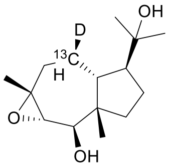	95.0%	0
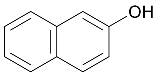	cytochrome p450monooxygenase enzyme cyp101b1	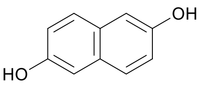	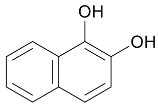	84.0%	0
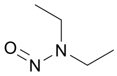	recombinant rat nadph-p450 reductase yeast glucose-6-phosphate dehydrogenase	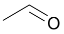	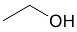	83.0%	2
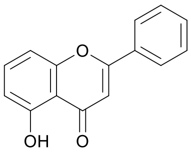	recombinant escherichia coli nadph-p450 reductase glucose 6-phosphate dehydrogenase	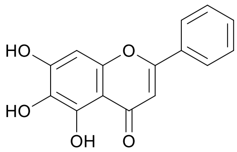	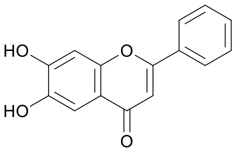	77.0%	4
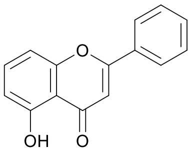	recombinant escherichia coli nadph-p450 reductase glucose 6-phosphate dehydrogenase	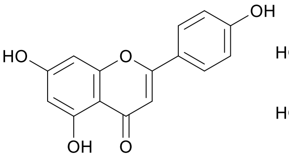	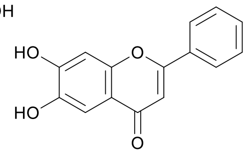	77.0%	4
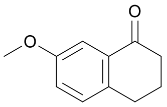	cytochrome p450monooxygenase p450rpmo	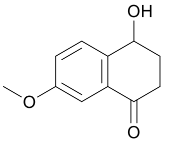	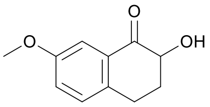	74.8%	0
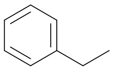	p450tol-glucose dehydrogenase	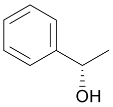	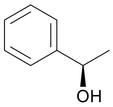	71.7%	0
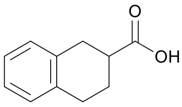	p450 peroxygenase oletje	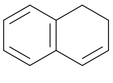	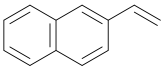	71.5%	0

Substrate	Enzymes	Database	Top 1 Prediction	Confidence Score	Rank
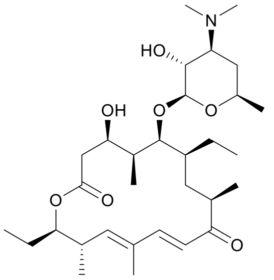	n-terminal mbp-tagged spinach ferredoxin reductase p450 monooxygenase mycci from micromonospora griseorubida	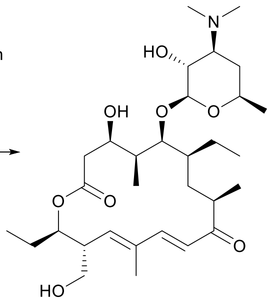	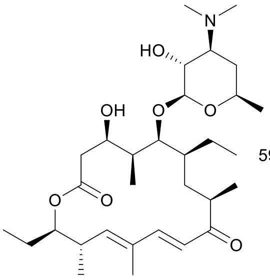	59.2%	2
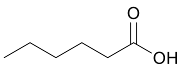	p450 peroxygenase from clostridium acetobutylicum 2-hydroxyisocaproate dehydrogenase from lactobacillus case i 2-hydroxyisocaproate dehydrogenase from lactobacillus confusus leucine dehydrogenase from bacillus cereus	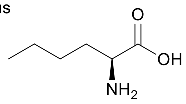	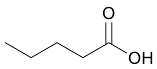	55.3%	0
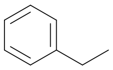	glucose dehydrogenasemonooxygenase cytochrome p450 lamo, t121p y385f m329l mutant	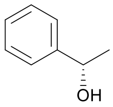	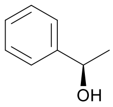	49.0%	2
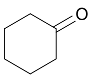	e. coli cells co-expressing cytochrome p450monooxygenase from bacillus megaterium with atd04 mutant and meso-butanedioldehydrogenase	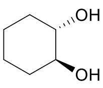	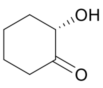	46.8%	0
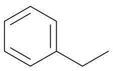	cytochrome p450monooxygenase s from amycolatopsisthermoflava	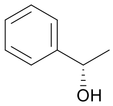	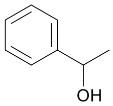	46.6%	0
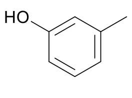	ferredoxin reductase 4-methylphenyl phosphate synthase p450 monooxygenase alcohol dehydrogenase aldehyde dehydrogenase phosphohydrolase	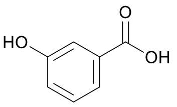	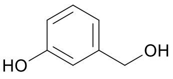	46.5%	0
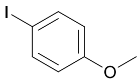	cytochrome p450monooxygenase p450 rpmo	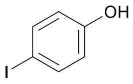	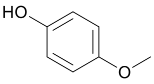	36.6%	2
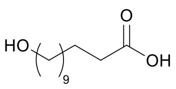	phosphite dehydrogenase oletje decarboxylase p450bm3 reductase domain fusion protein catalase	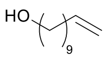	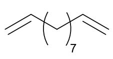	36.5%	0
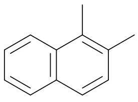	ferredoxin reductase p450monooxygenase cyp101b1 from a novosphingobium bacterium, wild type bovine liver catalase	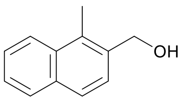	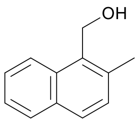	24.9%	2
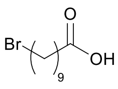	phosphite dehydrogenase oletje decarboxylase p450bm3 reductase domain fusion protein catalase	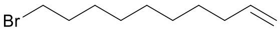	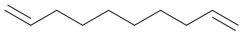	20.4%	0

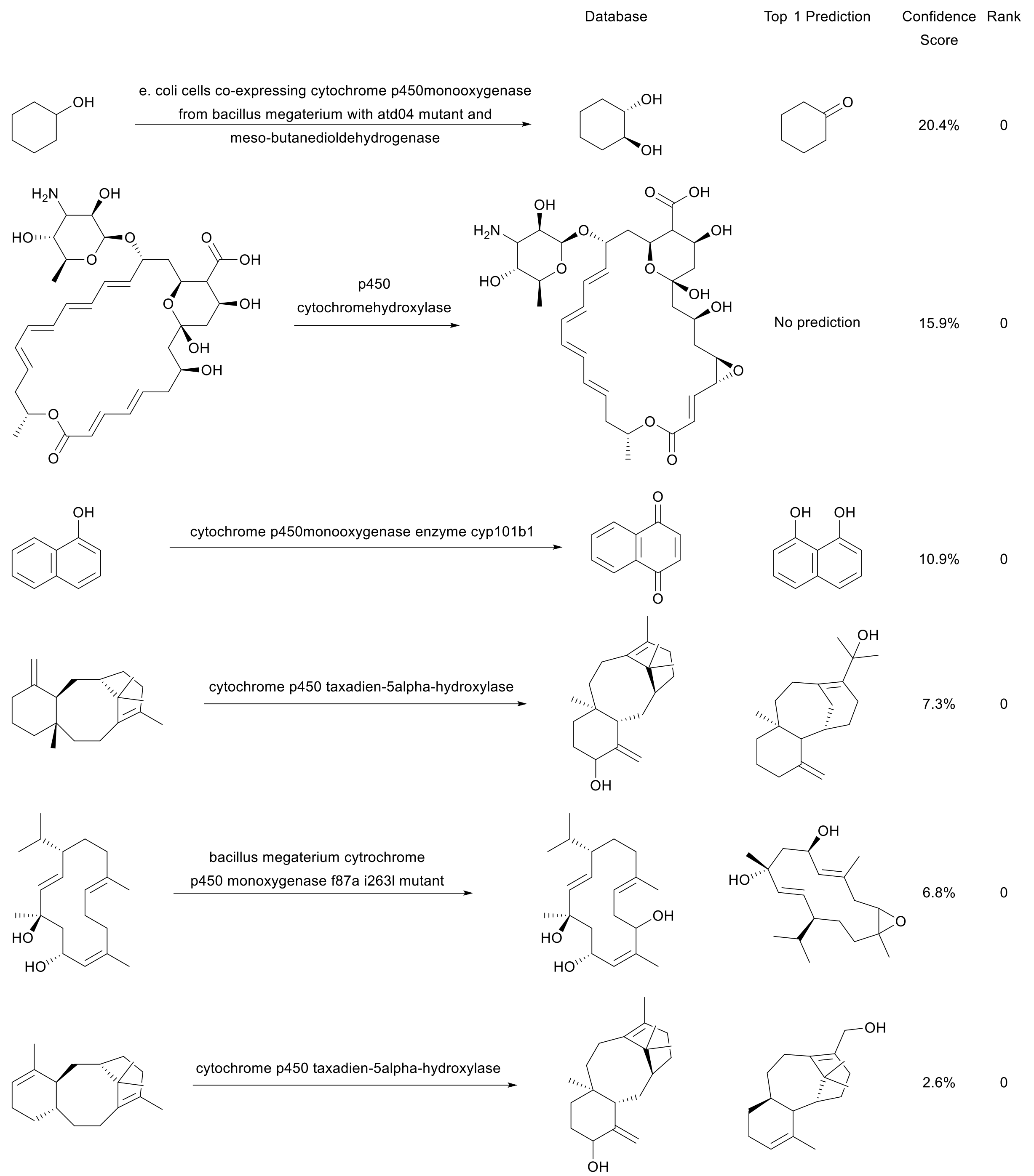

Figure S6. Every reaction from the test set containing "p450" in the sentence incorrectly predicted by the full sentence model. "rank" showing the rank of the correct prediction assigned by the model, "0" meaning that the model did not predict the correct product within the 5 first predictions. Reactions are sorted by decreasing confidence score.

Oxidase wild type (WT) and mutant (M).

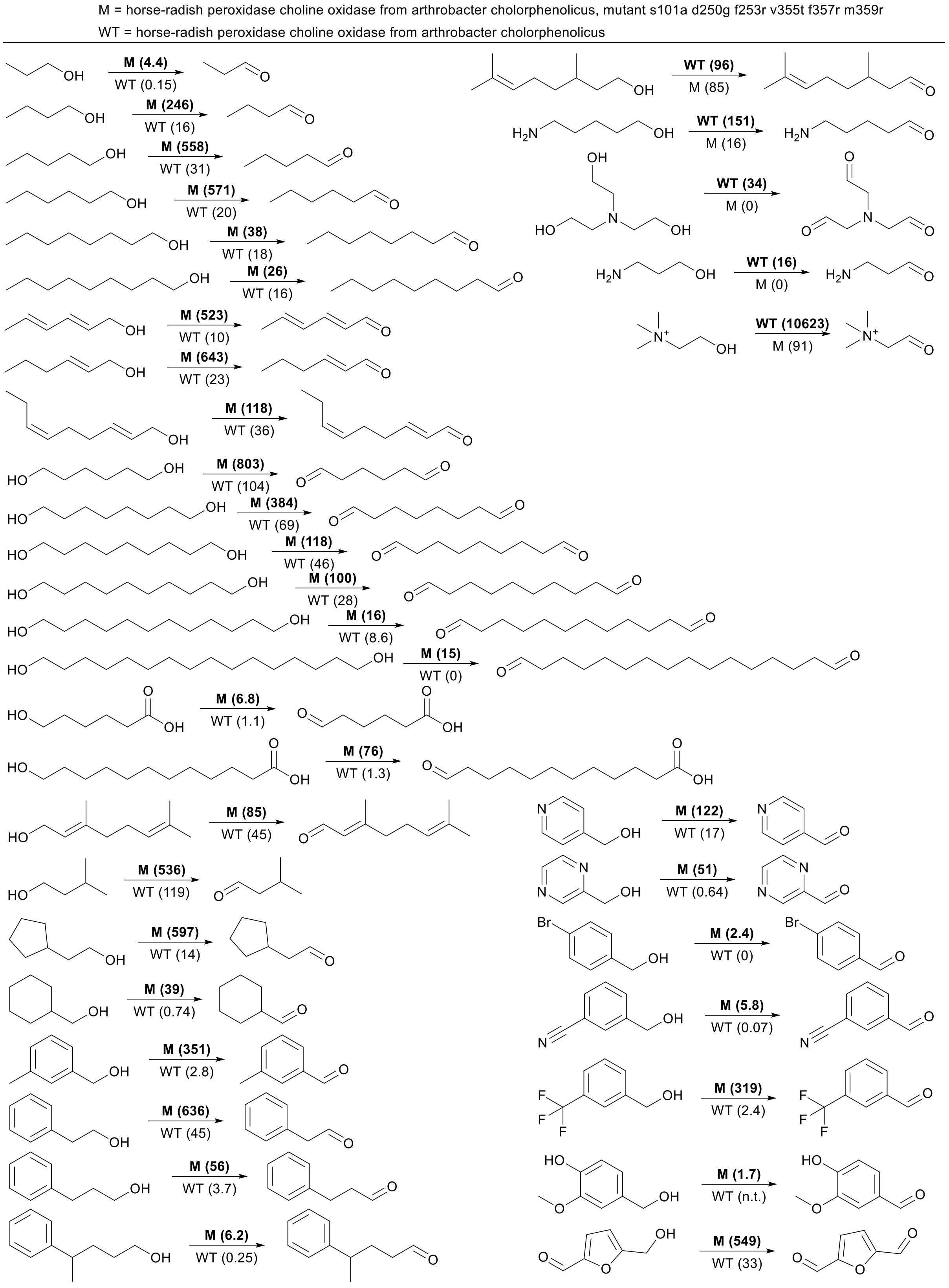

Figure S7. Reactions using the choline oxidase wild type (WT) and mutant (M) from Heath *et al.*[1] that were assigned to the training set. The numbers in parenthesis correspond to the specific activity of either the mutant or the wild type enzyme express in mU.mg^{-1}. (n.t. = not tested).

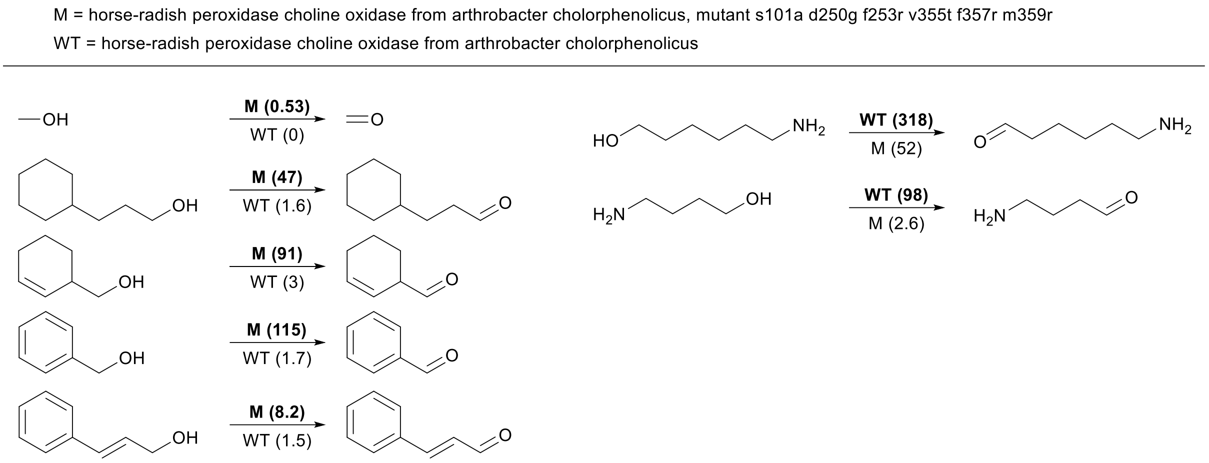

Figure S8. Reactions using the choline oxidase wild type (WT) and mutant (M) from Heath *et al.*[1] that were assigned to the validation set. The numbers in parenthesis correspond to the specific activity of either the mutant or the wild type enzyme express in mU.mg^{-1}.

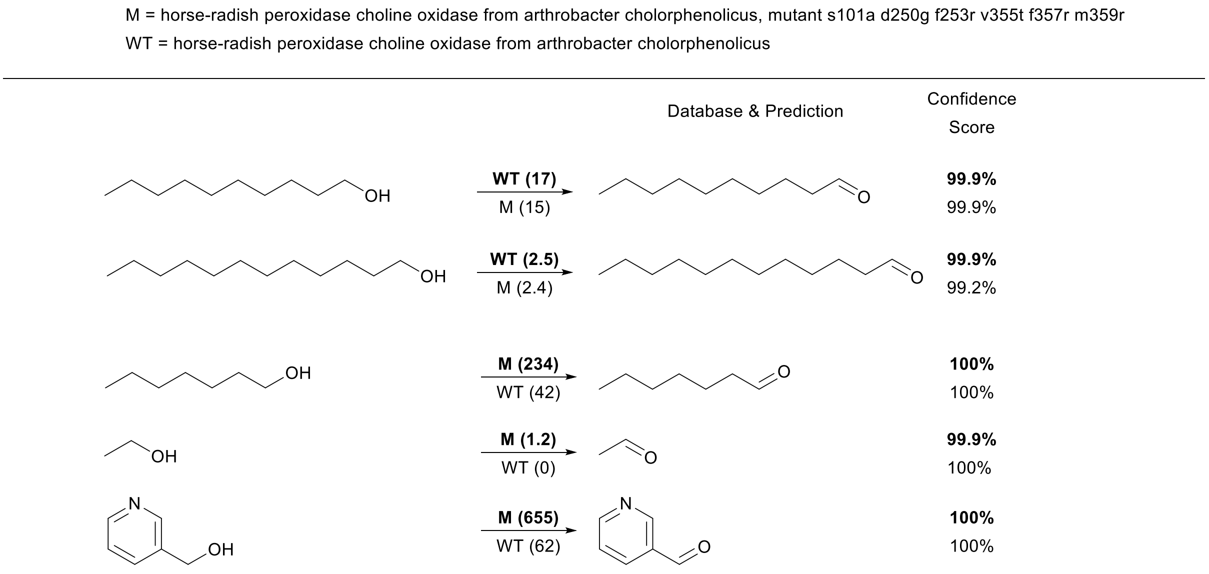

Figure S9. Reactions using the choline oxidase wild type (WT) and mutant (M) from Heath *et al.*[1] that were assigned to the test set. All reactions were predicted correctly. The numbers in parenthesis correspond to the specific activity of either the mutant or the wild type enzyme express in mU.mg^{-1}.

Screening of various substrates for the same sentences.

E = d-glucose dehydrogenase alcohol dehydrogenase ymr226c from saccharomyces cerevisiae
AD = alcohol dehydrogenase

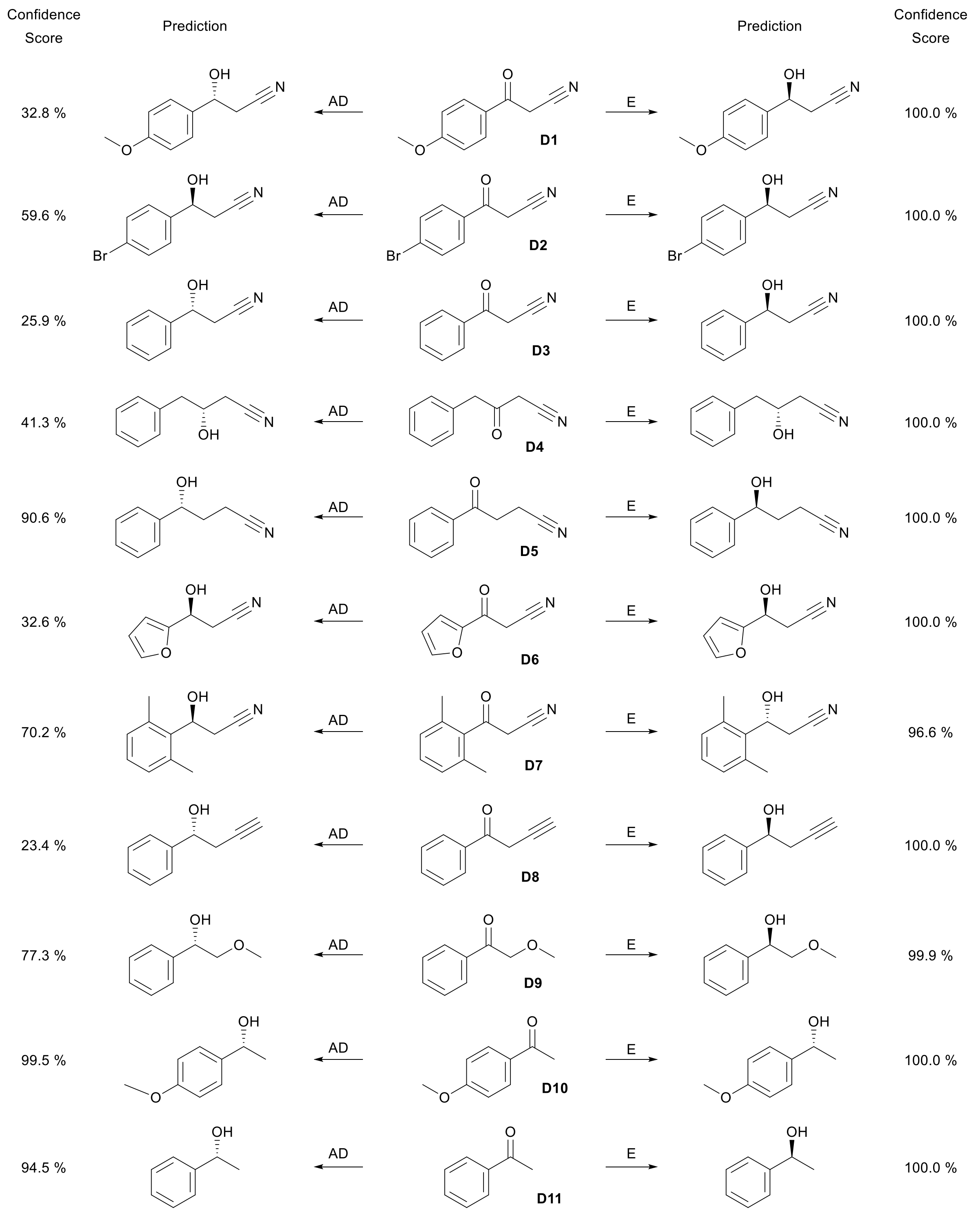

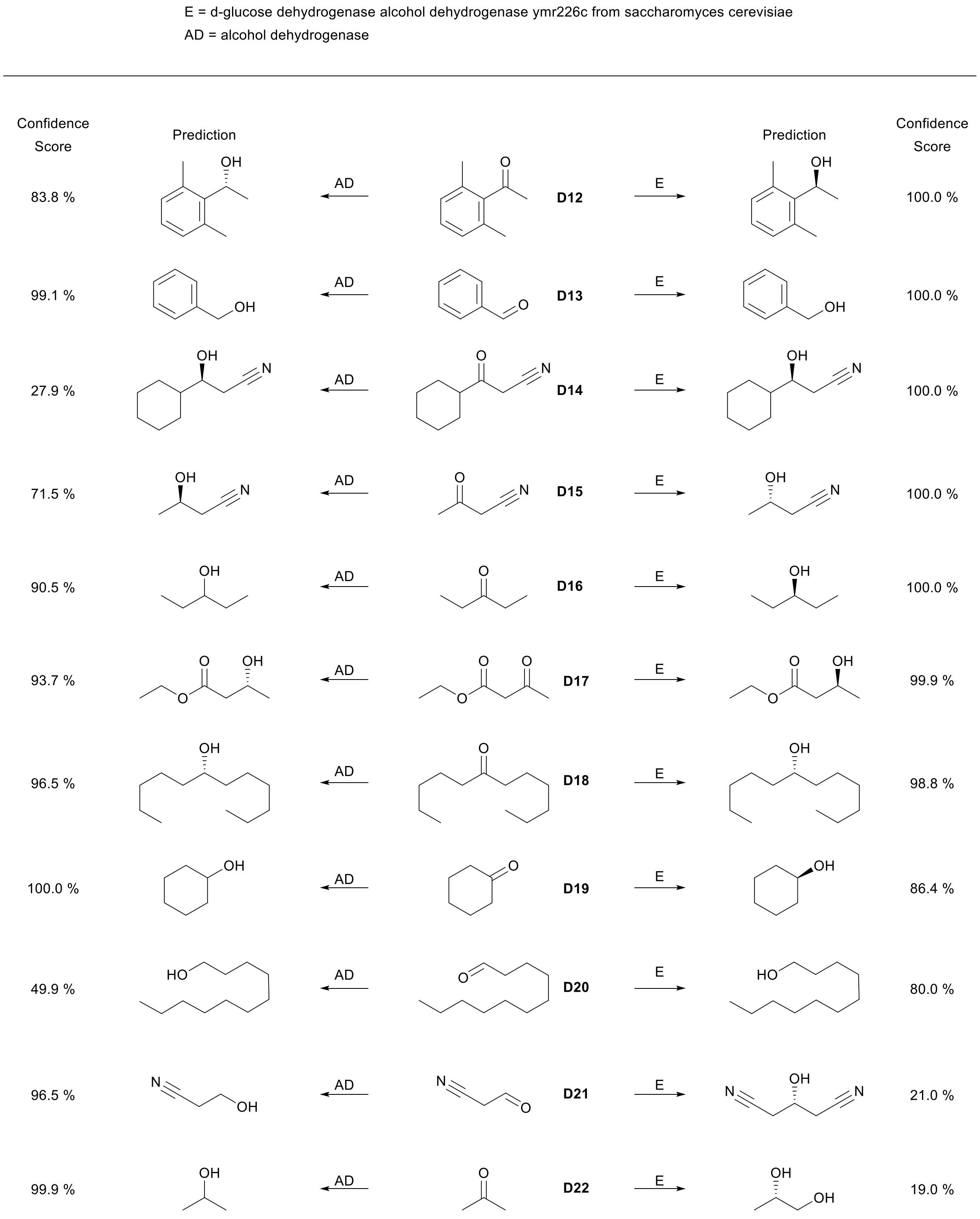

Figure S10. Various substrates tested on two sentences, a simple "alcohol dehydrogenase" (AD) and the "d-glucose dehydrogenase alcohol dehydrogenase ymr226c from *Saccharomyces cerevisiae*" (E). All substrates were derivatives from **D1** and **D2** which were present in the test set[2] and predicted correctly. Even though products from substrates **D16** and **D19** using enzyme "E" are not chiral, the model gave those chiral centers in the output SMILES ("CC[C@H](O)CC" for **D16**, "O[C@H]1CCCCC1" for **D19**).

Token frequencies analysis.

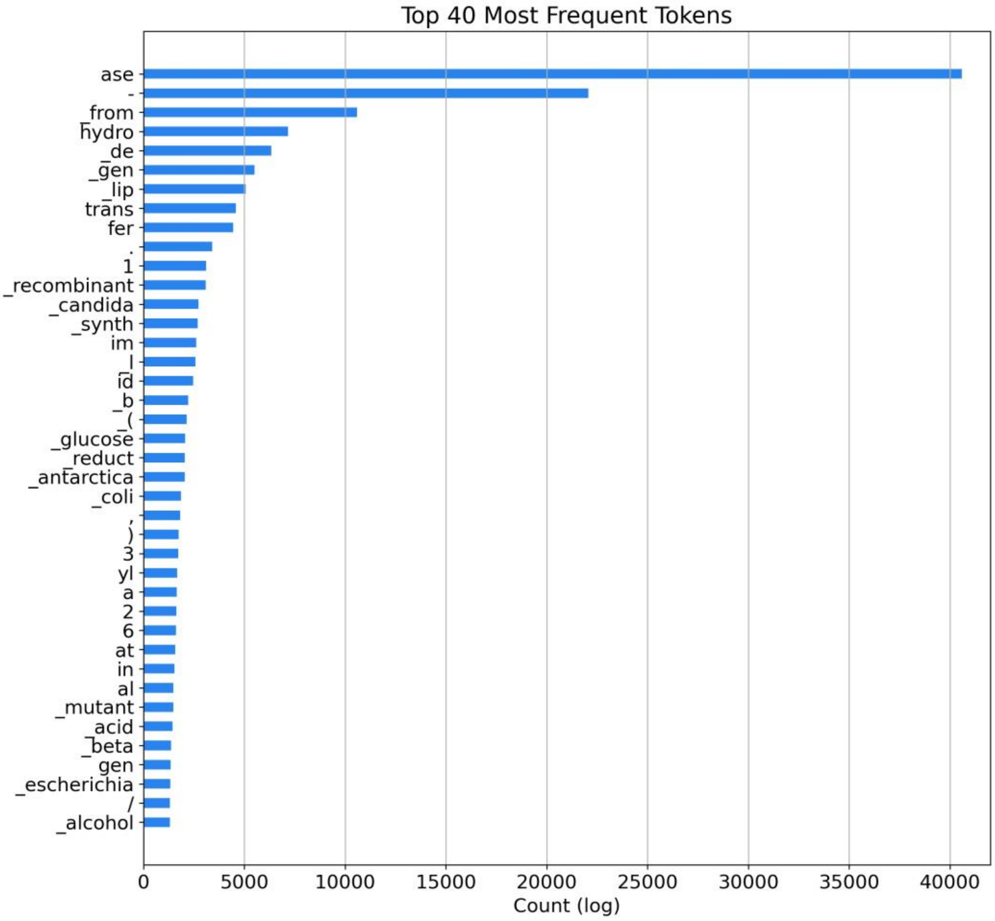

Figure S11. Top 40 most frequent tokens from the entire ENZR dataset.

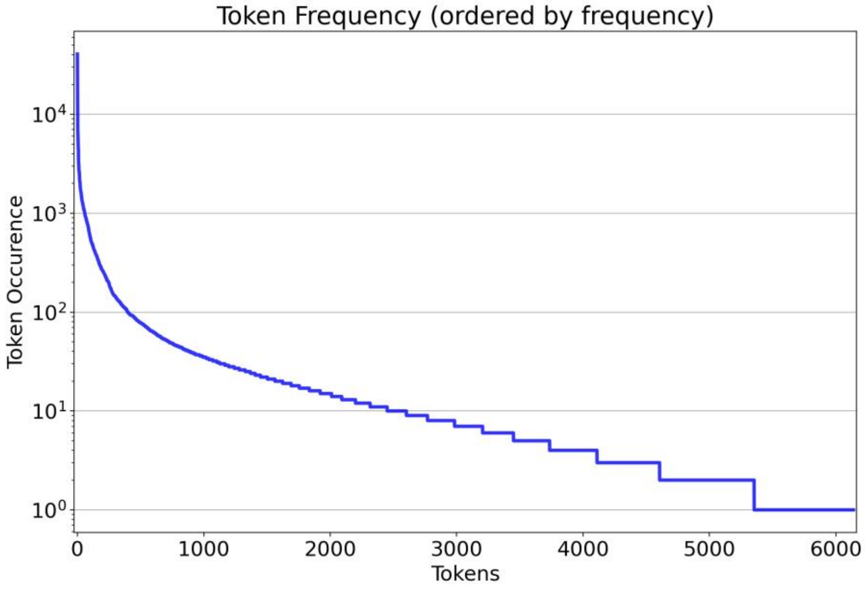

Figure S12. Power law distribution of the occurrence frequencies of all tokens in the ENZR sentences sorted by frequency (total of 6,139 tokens).

References

1 R. S. Heath, W. R. Birmingham, M. P. Thompson, A. Taglieber, L. Daviet and N. J. Turner, *ChemBioChem*, 2019, **20**, 276–281.

2 H. Ankati, D. Zhu, Y. Yang, E. R. Biehl and L. Hua, *J. Org. Chem.*, 2009, **74**, 1658–1662.
